# Prediction of Antifungal Activity of Antimicrobial Peptides by Transfer Learning from Protein Pretrained Models

**DOI:** 10.3390/ijms241210270

**Published:** 2023-06-17

**Authors:** Fernando Lobo, Maily Selena González, Alicia Boto, José Manuel Pérez de la Lastra

**Affiliations:** 1 Programa Agustín de Betancourt, Universidad de La Laguna, 38206 La Laguna, Tenerife, Spain; 2 Instituto de Productos Naturales y Agrobiología del CSIC, Avda. Astrofísico Fco. Sánchez, 3, 38206 La Laguna, Tenerife, Spainalicia@ipna.csic.es (A.B.); jm.perezdelalastra@csic.es (J.M.P.d.l.L.)

**Keywords:** antimicrobial peptides, antifungal peptides, transfer learning, machine learning

## Abstract

Peptides with antifungal activity have gained significant attention due to their potential therapeutic applications. In this study, we explore the use of pretrained protein models as feature extractors to develop predictive models for antifungal peptide activity. Various machine learning classifiers were trained and evaluated. Our AFP predictor achieved comparable performance to current state-of-the-art methods. Overall, our study demonstrates the effectiveness of pretrained models for peptide analysis and provides a valuable tool for predicting antifungal peptide activity and potentially other peptide properties.

## 1. Introduction

The emergence of antimicrobial resistance has posed a major threat to human and animal health [1], crop production, and food security [2]. Both the WHO and the FAO have claimed that urgent actions should be taken to face this challenge [1,2]. The development of new antimicrobials that induce little or negligible resistance has attracted much attention, particularly the discovery of novel host defense peptides and their synthetic analogues [3,4,5,6]. These peptides have a relatively small size (in general, less than 100 amino acids) and an amphiphilic nature, with a net positive charge that allows their anchoring to the negatively-charged bacterial membranes [7,8,9,10,11]. Many host defense peptides act by disrupting the pathogen membrane, but they are also capable of altering the nucleic acids, affecting the cell metabolism, and stimulating the host immune system, among other effects [3,4,5,6,12]. These multiple mechanisms of action greatly hinder the appearance of resistances [3,4,5,6,13].

In recent years, many variations in host defense peptides have been introduced to study their mechanism of action and to obtain derivatives with improved properties, such as higher efficacy or resistance to protease degradation, broader antimicrobial spectrum (or, on the contrary, a more selective action), fewer side effects (e.g., hemolytic activity), or lower production costs. Thus, truncated peptides or those with D- or unnatural L-residues, small and ultra-small peptides, or hybrid antimicrobial peptides have been described [3,4,5,6,7]. In general, a good balance between hydrophobic and cationic residues is necessary for strong antimicrobial activity and good selectivity [5]. The understanding of how physicochemical features play a key role in antimicrobial activity has attracted much interest from academia and industry [14].

In this context, computational design [15,16,17] and classification [17,18,19] methods play a crucial role in optimizing the sequence of antimicrobial peptides (AMPs). These methods enable the evaluation of antimicrobial activity based on the structural and amino acid sequence characteristics of peptides, bypassing the need for physical synthesis and testing [17].

Classification methods can be employed to develop predictive models that can classify peptides as antimicrobial or non-antimicrobial based on their sequence features. These models are trained on annotated datasets containing information about the antimicrobial activity of different peptides. By analyzing patterns and relationships in the data, classification algorithms can learn to distinguish between peptides with antimicrobial activity and those devoid of it, before their synthesis and experimental validation. This classification enables researchers to prioritize and focus their efforts on peptides with a higher likelihood of success, thereby saving time and resources in the drug discovery process [18].

By combining the power of rational peptide design with predictive modeling, researchers can efficiently navigate the vast sequence space of peptides and identify promising candidates for further experimental investigation [19]. This synergy between computational and experimental approaches holds great potential for accelerating the development of effective antimicrobial therapies to combat the growing threat of antibiotic resistance.

Today, evaluation of peptide antimicrobial activity relies heavily on machine learning (ML) tools. Initially, conventional ML algorithms [20] such as support vector machine (SVM) [21,22,23,24,25], random forest (RF) [26,27,28,29], k-nearest neighbors (kNN) [24,30,31], and multilayer perceptron (MLP) [32,33,34] were employed. These algorithms utilize the physicochemical and structural features of peptides, including hydrophobicity, net charge, isoelectric point, amino acid composition, and α, β, and turn propensity to predict their bioactivity. However, it is important to note that conventional ML methods necessitate a comprehensive understanding of the underlying mechanisms to accurately select the most appropriate features.

On the other hand, deep learning is an advanced machine learning approach that enables computers to automatically extract, analyze, and understand valuable information from raw data. This method has the ability to identify features that contain the most significant information while disregarding less relevant parameters [35].

Deep learning algorithms exhibit various architectures, including convolutional neural networks (CNNs), primarily utilized for image analysis. Additionally, recurrent neural networks (RNNs) are employed for analyzing sequential inputs such as text or biomolecule sequences [36,37]. However, recently developed transformers have recently surpassed RNNs. Unlike recursive analysis of sequential inputs, they can simultaneously analyze all tokens in the input [37].

**Figure 1:**
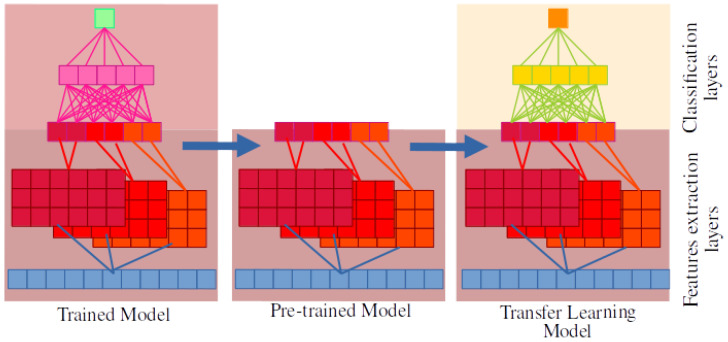
Overview of the transfer learning process. From an already trained model, the last layers (pink), are removed, leaving a pretrained model (red). Over this one, new layers (yellow) can be added to create a new model that takes advantage of the training of the original model.

Deep learning methods necessitate a substantial training dataset and intricate architecture design. However, these challenges can be overcome by implementing transfer learning strategies, which leverage pretrained models from other tasks [38]. Conceptually, this approach can be envisioned as utilizing a neural network that has already been trained for a specific task, in which the last layers responsible for classification are removed while retaining the initial layers responsible for feature extraction. New layers can then be added to construct a novel model tailored to a different task. Initially, the parameters of the pre-existing layers can be kept frozen, with only the parameters of the new layers requiring adjustment. Subsequently, if necessary, the remaining parameters can be fine-tuned to enhance accuracy (Figure 1).

To train these pretrained models, an extensive dataset is required. However, obtaining a reliable labeled dataset can be challenging, leading to the adoption of a self-supervised approach. For instance, in natural language processing (NLP), pretrained models have been trained to predict a subsequent token (such as a word, phrase, or punctuation symbol) based on the preceding tokens within a sentence. Since texts do not necessarily need to be labeled, models can be trained on vast amounts of text data, such as Wikipedia or Google Books.

For transfer learning to be effective, the pretrained model needs to have been trained on a diverse and representative dataset that captures the relevant patterns and characteristics of the target task. If the pretrained model is trained on a dataset that is too small or not representative of the target task, it may not capture the necessary information and may fail to transfer effectively.

Additionally, the quality of the data used for both pretraining and fine-tuning is crucial. Data with errors, inconsistencies, or biases can adversely affect the performance of the transfer learning model. Therefore, it is important to ensure that the training data, both for pretraining and fine-tuning, is of high quality, accurately labeled, and representative of the target task [39,40].

In the field of NLP, we can find models such as ELMo [41], which uses a bidirectional model with two RNNs to predict tokens based on surrounding context; GPT [42], which combines self-supervised training with supervised fine-tuning using a transformer architecture; and BERT [43], another transformer-based model, that predicts masked tokens inside a sentence to gain a deeper understanding of context.

These algorithms can also be extended to protein sequences, in which amino acids are used as tokens instead of words. In this context, self-supervised methods have been employed to generate pretrained models by predicting masked residues within proteins sourced from extensive databases like UniProt [44].

Some examples of those protein embedders, which have been assessed in this work, encompass Bepler [45], which utilizes a bidirectional LSTM neural network trained on the Pfam database via an ELMo-like self-supervised training approach; PlusRNN [46], a bidirectional recurrent neural network trained on Pfam, employing a combination of BERT-like self-supervised training and supervised same family prediction; SeqVec [47], another neural network based on BiLSTM, trained on the UniRef50 dataset using an ELMo-like self-supervised training methodology. ESM1b [48], a protein transformer model with approximately 650 million hyperparameters, trained on UniRef50 via a BERT-like self-supervised training paradigm; ProtTranBERTBFD [49], a transformer model with around 450 million hyperparameters, trained on the BFD dataset using a BERT-like self-supervised training approach; and ProtTransT5BFD [49], a larger transformer model with roughly 3 billion hyperparameters, trained on BFD utilizing a T5-like self-supervised training approach.

To illustrate their utility, Stärk et al. [50] developed a model that predicts protein localization based on the sequence, leveraging the pretrained ProtTransT5 model [49]. Furthermore, pretrained protein models have been utilized to predict properties of short peptides. For instance, Salem et al. [51] developed a model based on ProtTransBert [49] to predict the hemolytic activity of AMPs.

In the realm of peptide research, pretrained models can be integrated into hybrid models that combine DL methods with classical ML algorithms. For instance, Jiang et al. [52] developed models to predict peptide bitterness using pretrained RNNs, with their features subsequently fed into other ML algorithms like RF, SVM, or gradient boosting.

Our study is devoted to the development of models for predicting the antifungal efficacy of peptides using the six pretrained methods previously described. Existing literature on antifungal peptide (AFP) prediction covers mainly two approaches, classical quantitative structure–activity relationship (QSAR) descriptors and transfer learning methods.

Zhang et al. [53] recently presented an accurate AFP prediction model based on classical QSAR descriptors. Their methodology involves combining an antifungal activity classifier with four regression models to predict minimum inhibitory concentration (MIC) descriptors, which serve as feature extraction methods. The fusion of these five models enables the generation of an “Antifungal Index”, providing quantitative rankings for peptides. This index was utilized to screen a vast database of over three million peptides, facilitating the identification of the most promising candidates.

In contrast, transfer learning-based AFP predictors, such as Deep-AFPpred [54], employ a deep neural network that leverages the SeqVec pretrained model. In this approach, peptide sequences are transformed into per-residue embeddings, which are subsequently processed through a sequence of convolutional, pooling, recurrent, and dense layers. The resulting model exhibits exceptional performance but is limited to peptides with a maximum length of 30 residues, which corresponds to the input dimension of the convolutional layer.

In our proposed method, the embeddings of each residue were subjected to global average pooling to obtain a corresponding 1D vector representation of the peptide. Although this approach is simpler, we have found that these embeddings still retain the antifungal activity information of the peptides, making them amenable to analysis using classical machine learning algorithms. The resulting models exhibited comparable performance to the current state-of-the-art predictors of antifungal peptides.

## 2. Results and Discussion

**Figure 2:**
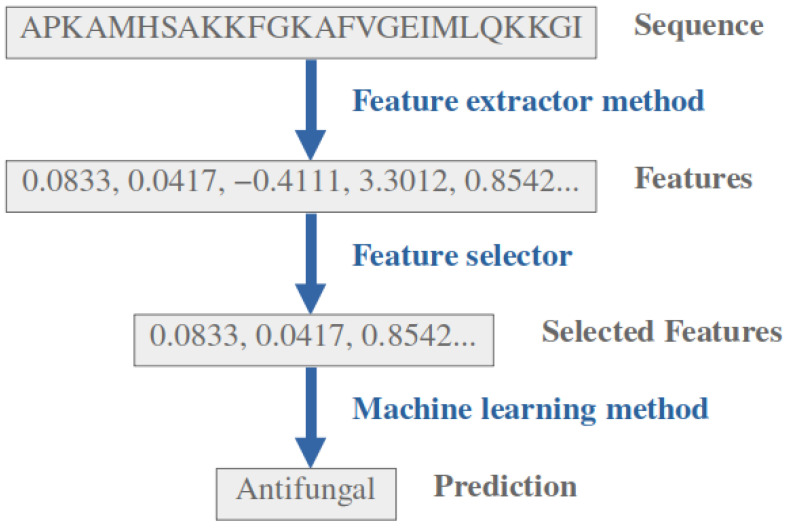
Antifungal predictors pipeline, consisting of three steps: (1) Sequences are converted to an array of features by a feature extractor method (pretrained protein model or QSAR descriptors); (2) the features array dimensionality is reduced by either most relevant features selection or principal components; (3) prediction of the antifungal activity using an ML algorithm.

Figure 2 illustrates the pipeline employed by the classifiers in our study to evaluate the antifungal activity of peptides. The models consist of a sequence-to-feature transformation system, followed by dimensionality reduction algorithms, and, finally, a machine learning classifier that predicts the antifungal activity based on the selected features.

To extract features from the peptide sequences, we evaluated six pretrained protein models (Bepler’s, SeqVec, PlusRNN, ESM1b, ProtTrans, and ProtT5). Additionally, we explored an alternative approach using a set of 76 QSAR descriptors for peptides.

For reducing the dimensionality of the features array, we explored multiple approaches in our study. This exploration included testing a principal component analysis (PCA) algorithm and three feature selection methods that aim to identify and retain the most relevant features for the prediction task (See Section 3). In parallel, we also developed predictors that do not involve reducing the input dimensionality. These predictors leverage the entire set of features without any dimensionality reduction step.

By examining both dimensionality reduction techniques and predictors without dimensionality reduction, we aimed to thoroughly investigate the effects of the different approaches on the performance of our models for predicting antifungal activity.

To perform the prediction, we employed various machine learning classifiers, including SVC, k-NN, MLP, logistic regression (LR), and RF. Additionally, we trained Stack models, which combine multiple methods and assign weights based on their prediction accuracy.

In total, we examined 210 combinations (7 feature extractors × 5 feature selectors × 6 machine learning algorithms). Each option was trained five times to ensure robust evaluation and to obtain reliable metrics.

To achieve our objective, we collected a dataset of peptides with antifungal and non-antifungal activity from the DBAASP database [8]. This database provides quantitative information on the antimicrobial activity of peptides. For our positive dataset, we selected peptides with a minimum inhibitory concentration (MIC) lower than 10 μM against any fungal species. Conversely, for our negative dataset, we selected peptides with MIC values greater than 100 μM against all microbial species tested. To ensure data quality, we removed redundant sequences using the CD-HIT script [55].

To minimize the influence of peptide length on predictions, we carefully curated both datasets to have the same range of peptide lengths. This approach helps to ensure fair and unbiased evaluations based on peptide lengths.

As is common in the training of machine learning classifiers, we divided the complete dataset into two sets: a training dataset consisting of 80% of the peptides, which was used to train the models, and a test dataset comprising the remaining 20% of the peptides, which served to evaluate the performance of the models. It is important to note that both datasets contained an equal number of positive and negative cases to maintain balance.

Hyperparameter tuning was conducted solely on the training dataset using a five-fold cross-validation strategy (See Section 3). This rigorous approach enabled us to optimize the performance of our models while mitigating the risk of overfitting and ensuring their generalizability. By reserving the test dataset solely for evaluation purposes, we could accurately assess the models’ performance on unseen data, providing a reliable measure of their effectiveness.

The quality of each method was evaluated using the following metrics:

$$\mathrm{Accuracy}:\;\frac{TP+TN}{TP+TN+FP+FN}$$

$$\mathrm{Precision}:\;\frac{TP}{TP+FP}$$

$$\mathrm{Recall}:\;\frac{TP}{TP+FN}$$

$$F1:\;2\frac{\mathrm{Precision}\times \mathrm{Recall}}{\mathrm{Precision}+\mathrm{Recall}}=\frac{2TP}{2TP+FP+FN}$$

$$\mathrm{MCC}:\;\frac{TP\cdot TN-FP\cdot FN}{\sqrt{\left(TP+FP\right)\left(TN+FN\right)\left(TP+FN\right)\left(TN+FP\right)}}$$

*TP*: True Positive; *TN:* True Negative; *FP*: False Positive; *FN*: False Negative.

**Figure 3:**
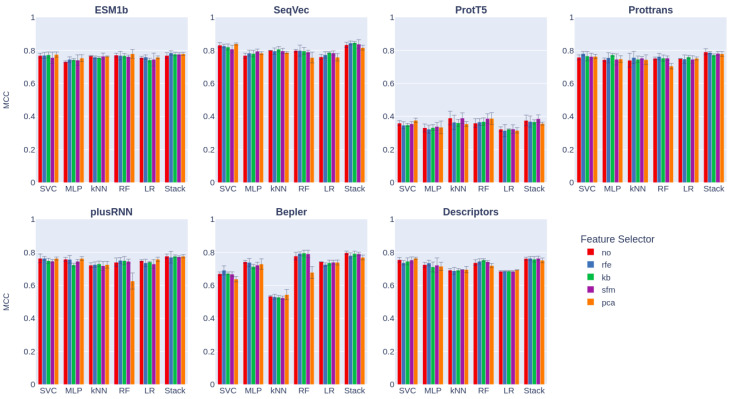
The Matthews correlation coefficient for each features extractor—features selector—classifier triad. Feature selectors were no: not perform any selection; rfe: recursive feature elimination; kb: select k best; sfm: select from model; pca: principal component analysis. Classifiers were: SVC: support vector classifier; MPL: multilayer perceptron; kNN: k-nearest neighbors; RF: random forest; LR: logistic regression; Stack: stacking of previous classifiers. Error bars show the corresponding 95% confidence intervals.

The Matthews correlation coefficient (MCC) was chosen as the primary metric for comparing and selecting the best model, as it is a reliable measure when comparing methods trained on the same dataset [56]. The mean MCC values for each extractor–selector–classifier triad are depicted in Figure 3. Detailed metrics for each model can be found in Supporting Information Table S3.

Our analysis revealed that reducing the input dimensionality did not significantly impact the quality of the classifiers, regardless of the feature selection algorithm employed. A three-way analysis of variance (ANOVA) demonstrated that the feature selector variable yielded a p-value higher than 0.05 across all metrics. However, it is worth noting that a noticeable increase in performance was observed when the number of features was reduced. This result highlights the effectiveness of carefully selecting features to enhance model performance without sacrificing accuracy.

A comprehensive overview of MCC values for all feature extractor–classifier algorithm pairs, averaging across all feature selector techniques, can be found in Table 1. Additional metrics data can be accessed in the Supporting Information Table S4.

Among the feature extractors tested, SeqVec exhibited the best performance, surpassing heavier pretrained models such as ProtT5. Additionally, SVC demonstrated the highest metrics among all the machine learning algorithms. Stacking SVC models with other classifiers only resulted in a slight improvement in prediction efficiency.

Based on the obtained results, we have chosen a lead model for our study, which consists of a pipeline utilizing SeqVec as the feature extractor, followed by a k-Best feature selector, and ending with a support vector classifier. The reason for selecting k-Best as the feature selection algorithm is that it effectively reduces the input dimensionality and improves the model’s performance. This model has been trained and uploaded to https://huggingface.co/spaces/Flobopal/AFPtransferPred (accessed in both cases on 15 May 2023). The model can also be accessed at https://selectfight.org/afptransferpred/ (accessed in both cases on 15 May 2023). It is important to note that our method provides results that are competitive with those of existing approaches. Based on their given metrics, we compared the performance of our technique with those of other available methods (Table 2) and assessed them using our test dataset.

We acknowledge that Zhang’s method [53] based on classical QSAR descriptors demonstrated a high performance, as reported in their paper. However, it is crucial to consider the potential overlap between their training set and our test set, as both datasets include sequences from the DBAASP database. Upon investigation, we discovered that 75% of the sequences in our test dataset are also present in their dataset, as indicated in their GitHub repository (https://github.com/JinZhangLab/antifungal, accessed on 15 May 2023).

To address this issue and ensure a fair comparison, we took the necessary steps to remove the overlapping sequences from our test dataset. By doing so, we were able to evaluate our method’s performance more accurately. As a result, our method demonstrated a significantly higher performance when the test dataset was adjusted accordingly (compare rows marked as d in Table 2).

Similarly, Deep-AFPpred, [54], showed comparable performance to our work. However, it is worth noting that Deep-AFPpred is limited to accepting peptides with a maximum length of 30 amino acids.

In contrast, AntiFP [57] showed less favorable metrics in their evaluation. Indeed, the differences in the construction of positive and negative datasets between AntiFP’s approach and ours can contribute to the observed variation in performance metrics. AntiFP’s positive dataset was composed of peptides that are listed in the DRAMP database [10] as antifungal, without considering the quantitative activity of these peptides. In contrast, our positive dataset was carefully curated to include peptides with specific MIC or IC values against various antifungal strains, ensuring a quantitative measure of their antifungal activity.

Furthermore, AntiFP’s negative dataset consisted of a mixture of random peptides and peptides from the DRAMP database with activities other than antifungal. While this approach aims to create a diverse negative dataset, it may inadvertently include peptides that possess antifungal properties but were not specifically annotated as such in the database. This inclusion can introduce noise and reduce the discriminative power of the model.

By carefully curating our positive and negative datasets based on quantitative activity measurements and stringent criteria, we aimed to ensure a more accurate and reliable assessment of peptide antifungal activity. This meticulous dataset construction, combined with the utilization of advanced machine learning techniques and the integration of pretrained models, contributes to the improved performance observed in our method compared to AntiFP.

It is important to consider these differences in dataset construction when comparing the performance of different models. The specific criteria used for dataset creation can significantly impact the model’s ability to accurately predict antifungal activity.

## 3. Materials and Methods

### 3.1. Dataset Preparation

The dataset used in our study was obtained from the DBAASP database [8]. The positive dataset consisted of peptides that exhibited a minimum inhibitory concentration (MIC) or IC90 value lower than 10 μM, or an IC50 value lower than 2 μM, against fungal species belonging to any fungal genus such as *Fusarium*, *Candida*, *Botrytis*, *Cryptococcus*, *Aspergillus*, *Saccharomyces*, *Pichia*, *Batrachochytrium*, *Neurospora*, *Didymella*, *Leptosphaeria*, *Phytophthora*, *Verticillium*, *Fulvia*, or *Alternaria*. On the other hand, the negative dataset included sequences with an MIC or IC90 value higher than 100 μM, or an IC50 value higher than 20 μM, against all microbial species they were tested against. To remove redundant sequences, we utilized the CD-HIT script [55], which removed sequences that differed by less than three residues. Both the positive and negative datasets were further divided into subsets based on specific sequence lengths (e.g., 10–20, 21–30, 31–40, and so on). The final datasets were created by adjusting the size of each subset so that pairs of positive and negative subsets with the same sequence length range had equal sizes. If one subset was larger than the other, the necessary number of sequences were randomly eliminated from the dataset using the sample function from Python’s random library. Finally, the positive and negative datasets were divided into their respective training and test datasets. The test datasets were created by randomly selecting 20% of the sequences from the original datasets using the random sample function, while the training datasets comprised the remaining sequences from each original dataset.

### 3.2. Features Extraction

Pretrained models were applied using the bio_embeddings library, according to the developers’ suggested protocol [58]. From this library, we used the embedders: BeplerEmbedder (Bepler), PLUSRNNEmbedder (PlusRNN), SeqVecEmbedder (SeqVec), ESM1bEmbedder (ESM1b), ProtTransBertBFDEmbedder (Prottrans), and ProtTransT5BFDEmbedder (ProtT5).

ProtT5 was applied in a Google Colab notebook, using TPU as hardware accelerator. The rest of the models were run on a personal computer with a 16 nucleus 11th Gen Intel^®^ Core™ i7-11800H @ 2.30 GHz, 16 GB RAM, and an 8 GB NVIDIA GeForce RTX 3050 Mobile GPU.

In parallel, we also obtained a series of 76 QSAR descriptors for peptides using the descriptors function from the Python peptides library [59].

### 3.3. Features Selection

To identify the most relevant features, we utilized four algorithms from the scikit-learn [60] Python library:

- PCA (Principal Component Analysis): This method performs dimensionality reduction by transforming the original features into a new set of uncorrelated variables called principal components. We used Minka’s maximum likelihood estimation (MLE) to determine the optimal number of components.
- SelectFromModel: In this approach, we initially trained a random forest classifier using all the available data. Then, we selected features with an absolute importance greater than 0.0001, as determined by the classifier.
- RFECV (Recursive Feature Elimination with Cross-Validation): This method recursively eliminates the least relevant feature based on the performance of a random forest model. The evaluation is conducted using a 5-fold cross-validation, and the final set of selected features is determined by the iteration that yields the best cross-validation score.
- SelectKBest: This approach selects the K best features based on the results of an ANOVA F-test between the features and the corresponding labels. The number of selected features is set to one-fifth of the dataset size.
- Additionally, we also trained and tested pipelines that utilized the full feature arrays without any dimensionality reduction.

### 3.4. Models Training

The scikit-learn library in Python was utilized to train the models in our study. We experimented with various classifiers available in scikit-learn: SVC, KneighborsClassifier (kNN), RandomForestClassifier (RF), LogisticRegression (LR), and MultilayerPerceptronClassifier (MLP).

To optimize the hyperparameters of these classifiers, we employed a cross-validation grid search strategy using the GridSearchCV function provided by scikit-learn. This approach involves randomly dividing the training dataset into five subsets, of which four subsets are used for training the model with a specific hyperparameter configuration, and the remaining subset is used for evaluation. This process is repeated five times, with each subset serving as the validation set once. By using this strategy, hyperparameters could be tuned using only the training dataset, ensuring unbiased evaluation. Supporting Information Table S1 contains hyperparameters optimized for each algorithm.

**Figure 4:**
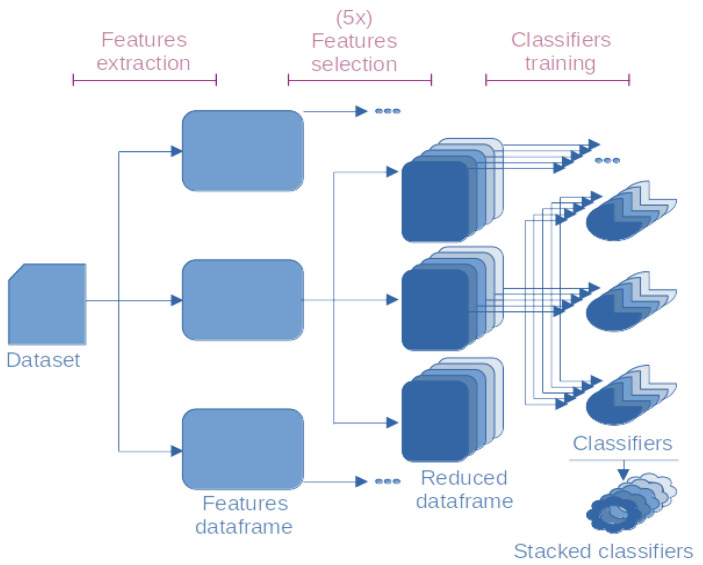
Three-step approach for model optimization. First, the peptide sequences dataset was transformed into corresponding feature vectors using a feature extractor. Then, a feature selector was applied five times to the feature vectors to identify the most relevant features. Finally, a classifier was trained on the selected features to predict the antimicrobial activity of the peptides. Bullets indicate that the same process was applied to all dataframes.

To streamline the search for the best model, we followed a three-step approach, as shown in Figure 4. Firstly, we applied each feature selection method to transform the sequence datasets into corresponding feature dataframes. Since the transformations are deterministic, this step only needed to be performed once for each method. Next, each feature selection algorithm was applied five times to each dataframe, resulting in reduced dataframes and the corresponding feature selector functions. These analyses were performed with random seeds, generating five different results for each application of the algorithm. Finally, each reduced dataframe was provided to each of the five classification algorithms for training the final classifiers. Additionally, the five classifiers trained from each reduced dataset were stacked using the StackingClassifier class, which combines their predictions to obtain an ensemble model.

Supporting Information Tables S2 and S3 show the best hyperparameters found for each model and its metrics, respectively. Table S4 shows the metrics for each features extractor–classifier pair, averaging between all feature selection models.

The final model was then trained as a scikit-learn Pipeline object, incorporating the best steps and parameters observed during the optimization phase. In the classification step, the probability attribute was set to True, enabling the model to output the predicted probabilities as likelihood values between 0 and 1 for the bioactivity of the peptide. The model was saved as a binary file using the pickle Python library and uploaded to the HuggingFace repository. Furthermore, a web server service using HuggingFace Space was developed, allowing users to access the model at https://selectfight.org/afptransferpred/ (accessed on 15 May 2023).

### 3.5. Evaluation

Metrics were calculated using the metrics module from the scikit-learn library. For the optimization phase, accuracy_score, precision_score, recall_score, f1_score, and matthews_corrcoef functions were employed to calculate the accuracy, precision, recall, and F1 and MCC scores, respectively.

In addition to these metrics, for the final model and the comparison with other AFP models, the roc_auc_score function was employed to calculate the area under the receiver operating characteristic (ROC) curve. This metric provides a measure of the model’s performance in terms of the trade-off between the true positive rate and the false positive rate.

### 3.6. Comparison with Other Available Models

The test dataset, appropriately sampled to meet the peptide length requirements of each model, was subjected to prediction analysis using Zhang’s [61], Deep-AFPpred [62], and AntiFP [63] web servers. The predictions obtained from these external servers were meticulously scrutinized and compared with the performance of our own model, as discussed in the preceding sections. This comparative analysis enabled us to assess the concordance and consistency between the predictions generated by these existing models and the results obtained from our developed model.

## 4. Conclusions

In this study, we have explored various pretrained protein models as feature extractors for predicting the antifungal activity of peptides. The models we developed have demonstrated similar performance to existing state-of-the-art methods, which utilize more complex architectures or classical quantitative structure–activity relationship (QSAR) descriptors.

During our evaluation, we also compared our models with other AFP predictors using our test dataset. We found that Zhang’s method and the DeepAFP server are reliable models for predicting the antifungal activity of peptides, while the AntiFP server showed limited prediction capability. In addition, our model can be used for peptides longer than 30 amino acids, a limitation noted with the DeepAFP server.

Overall, this work contributes to the field of peptide-based antimicrobial activity prediction by demonstrating the effectiveness of pretrained models and feature selection techniques. The pipeline employed in this project can be easily adapted to predict other peptide properties, such as antiviral, antibacterial, or hemolytic activities. By utilizing different datasets and adjusting the training process, it is possible to develop models for various peptide-related applications.

The model with the best performance has been uploaded, providing an alternative tool to existing AFP predictors. This model can be used by users and researchers to make predictions about the antifungal properties of peptides.

## Figures and Tables

**Table 1:** MCC for each feature extractor and ML algorithm pairs, averaging all feature selection methods.

	LR	RF	kNN	MLP	SVC	Stacking
SeqVec	0.773 ± 0.009	0.788 ± 0.013	0.797 ± 0.007	0.782 ± 0.008	0.825 ± 0.009	0.836 ± 0.009
Prottrans	0.751 ± 0.007	0.745 ± 0.013	0.748 ± 0.013	0.753 ± 0.011	0.766 ± 0.009	0.782 ± 0.006
Bepler	0.736 ± 0.006	0.766 ± 0.027	0.532 ± 0.008	0.729 ± 0.010	0.667 ± 0.012	0.786 ± 0.008
ESM1b	0.752 ± 0.009	0.769 ± 0.008	0.762 ± 0.005	0.742 ± 0.009	0.768 ± 0.009	0.777 ± 0.006
Descriptors	0.687 ± 0.002	0.740 ± 0.008	0.692 ± 0.006	0.721 ± 0.012	0.751 ± 0.008	0.759 ± 0.006
plusRNN	0.742 ± 0.009	0.722 ± 0.030	0.724 ± 0.008	0.749 ± 0.010	0.757 ± 0.008	0.774 ± 0.007
ProtT5	0.320 ± 0.009	0.374 ± 0.013	0.372 ± 0.016	0.332 ± 0.011	0.357 ± 0.009	0.371 ± 0.011

**Table 2:** Comparison between this work and other available AFP predictors.

		ROC AUC	Accuracy	Precision	Recall	F1	MCC	Ref.
This work	^a^	0.97	0.90	0.90	0.90	0.90	0.81	
	^b^	0.97	0.90	0.90	0.89	0.90	0.79	
	^c^	0.97	0.90	0.91	0.89	0.90	0.81	
	^d^	0.97	0.88	0.89	0.68	0.77	0.71	
Zhang et al.	^e^	0.95	0.89	0.90	0.89	0.89	0.79	[53]
	^a^	0.98	0.94	0.93	0.94	0.94	0.88	
	^d^	0.78	0.80	0.64	0.64	0.64	0.50	
Deep-AFPpred	^e^	0.98	0.94	0.95	0.93	0.94	0.89	[54]
	^b^	0.94	0.87	0.85	0.91	0.88	0.75	
AntiFP	^e^	0.92	0.85	-	0.85	-	0.69	[57]
	^c^	0.58	0.56	0.62	0.49	0.51	0.14	

^a^ Evaluated with this work’s full validation dataset; ^b^ Evaluated with this work’s <30 amino acid length sequences dataset; ^c^ Evaluated with this work’s <50 amino acid length sequences dataset; ^d^ using a dataset in which the sequences that appear in Zhang’s dataset were removed; ^e^ according to its publication.

## Data Availability

All sequences used in this work were downloaded from DBAASP database [8]. Information about the hyperparameters and metrics can be found in the Supplementary Material. Final Model can be accessed at https://selectfight.org/afptransferpred/ (accessed on 15 May 2023) or https://huggingface.co/spaces/Flobopal/AFPtransferPred (accessed on 15 May 2023).

## Supplementary Materials

The following supporting information can be downloaded at: https://www.mdpi.com/article/10.3390/ijms241210270/s1.

## References

[1] Antimicrobial Resistance, FAO-United Nations. http://www.fao.org/antimicrobial-resistance/en/.

[2] Antimicrobial Resistance, WHO-United Nations. https://www.who.int/health-topics/antimicrobial-resistance.

[3] Wang G. (2017). Antimicrobial Peptides: Discovery, Design and Novel Therapeutic Strategies.

[4] Lobo F., Boto A. (2022). Host-defense peptides as new generation phytosanitaries: Low toxicity and low induction of antimicrobial resistance. Agronomy.

[5] Boto A., de la Lastra J.M.P., González C.C. (2018). The Road from Host-Defense Peptides to a New Generation of Antimicrobial Drugs. Molecules.

[6] de Souza Cândido E., Cardoso M.H.S., Sousa D.A., Viana J.C., de Oliveira-Júnior N.G., Miranda V., Franco O.L. (2014). The use of versatile plant antimicrobial peptides in agribusiness and human health. Peptides.

[7] Kastin A.J. (2006). Handbook of Biologically Active Peptides.

[8] Antimicrobial Peptide Database-DBAASP. https://dbaasp.org/home.

[9] Antimicrobial Peptide Database-APD. https://aps.unmc.edu/.

[10] Data Repository of Antimicrobial Peptides-DRAMP. http://dramp.cpu-bioinfor.org/.

[11] Plant Antimicrobial Peptides-PhytAMP. http://phytamp.pfba-lab-tun.org/main.php.

[12] Yount N.Y., Yeaman M.R. (2005). Immunocontinuum: Perspectives in antimicrobial peptide mechanisms of action and resistance. Protein Pep. Lett..

[13] Fleitas O., Franco O.L. (2016). Induced Bacterial Cross-Resistance towards Host Antimicrobial peptides: A worrying phenomenon. Front. Microbiol..

[14] Pushpanathan M., Pooja S., Gunasekaran P., Rajendhran J. (2016). Critical Evaluation and Compilation of Physicochemical Determinants and Membrane Interactions of MMGP1 Antifungal Peptide. Mol. Pharm..

[15] Lee E.Y., Lee M.W., Fulan B.M., Ferguson A.L., Wong G.C.L. (2017). What can machine learning do for antimicrobial peptides, and what can antimicrobial peptides do for machine learning?. Interface Focus.

[16] Fjell C.D., Hiss J.A., Hancock R.E.W., Schneider G. (2012). Designing antimicrobial peptides: Form follows function. Nat. Rev. Drug Discov.

[17] Cardoso M.H., Orozco R.Q., Rezende S.B., Rodrigues G., Oshiro K.G.N., Cândido E.S., Franco O.L. (2020). Computer-Aided Design of Antimicrobial Peptides: Are We Generating Effective Drug Candidates?. Front. Microbiol..

[18] Yan J., Cai J., Zhang B., Wang Y., Wong D.F., Siu S.W.I. (2022). Recent Progress in the Discovery and Design of Antimicrobial Peptides Using Traditional Machine Learning and Deep Learning. Antibiotics.

[19] Aronica P.G.A., Reid L.M., Desai N., Li J., Fox S.J., Yadahalli S., Essex (2021). J.W.; Verma, C.S. Computational Methods and Tools in Antimicrobial Peptide Research. J. Chem. Inf. Model..

[20] Dara S., Dhamerchela S., Jadaw S.S., Babu C.H.M., Ahsan M.J. (2022). Machine Learning in Drug Discovery: A Review. Artif. Intell. Rev..

[21] Chang L., Mondal A., Perez A. (2022). Towards rational computational peptide design. Front Bioinform..

[22] Porto W.F., Pires A.S., Franco O.L. (2012). CS-AMPPred: An Updated SVM Model for Antimicrobial Activity Prediction in Cysteine-Stabilized Peptides. PLoS ONE.

[23] Meher P.K., Sahu T.K., Saini V., Rao A.R. (2017). Predicting antimicrobial peptides with improved accuracy by incorporating the compositional, physico-chemical and structural features into Chou’s general PseAAC. Sci. Rep..

[24] Kavousi K., Bagheri M., Behrouzi S., Vafadar S., Atanaki F.F., Lotfabadi B.T., Ariaeenejad S., Shockravi A., Moosavi-Movahedi A.A. (2020). IAMPE: NMR-Assisted Computational Prediction of Antimicrobial Peptides. J. Chem. Inf. Model..

[25] Xiao X., Shao Y.T., Cheng X., Stamatovic B. (2021). iAMP-CA2L: A new CNN-BiLSTM-SVM classifier based on cellular automata image for identifying antimicrobial peptides and their functional types. Brief. Bioinform..

[26] Bhadra P., Yan J., Li J., Fong S., Siu S.W.I. (2018). AmPEP: Sequence-based prediction of antimicrobial peptides using distribution patterns of amino acid properties and random forest. Sci. Rep..

[27] Chung C.R., Jhong J.H., Wang Z., Chen S., Wan Y., Horng J.T., Lee T.Y. (2020). Characterization and identification of natural antimicrobial peptides on different organisms. Int. J. Mol. Sci..

[28] Xu J., Li F., Leier A., Xiang D., Shen H.H., Marquez Lago T.T., Li J., Yu D.J., Song J. (2021). Comprehensive assessment of machine learning-based methods for predicting antimicrobial peptides. Brief. Bioinform..

[29] Tripathi V., Tripathi P. (2020). Detecting antimicrobial peptides by exploring the mutual information of their sequences. J. Biomol. Struct. Dyn..

[30] Sharma R., Shrivastava S., Singh S.K., Kumar A., Saxena S., Singh R.K. (2021). AniAMPpred: Artificial intelligence guided discovery of novel antimicrobial peptides in animal kingdom. Brief. Bioinform..

[31] Xiao X., Wang P., Lin W.Z., Jia J.H., Chou K.C. (2013). iAMP-2L: A two-level multi-label classifier for identifying antimicrobial peptides and their functional types. Anal. Biochem..

[32] Ahmad A., Akbar S., Khan S., Hayat M., Ali F., Ahmed A., Tahir M. (2021). Deep-AntiFP: Prediction of antifungal peptides using distanct multi-informative features incorporating with deep neural networks. Chemom. Intell. Lab. Syst..

[33] Timmons P.B., Hewage C.M. (2021). ENNAACT is a novel tool which employs neural networks for anticancer activity classification for therapeutic peptides. Biomed. Pharmacother..

[34] Timmons P.B., Hewage C.M. (2021). ENNAVIA is a novel method which employs neural networks for antiviral and anti-coronavirus activity prediction for therapeutic peptides. Brief. Bioinform..

[35] Zhang L., Tan J., Han D., Zhu H. (2017). From machine learning to deep learning: Progress in machine intelligence for rational drug discovery. Drug Discov. Today.

[36] Jing Y., Bian Y., Hu Z., Wang L., Xie X.Q. (2018). Deep Learning for Drug Design: An Artificial Intelligence Paradigm for Drug Discovery in the Big Data Era. AAPS J..

[37] Yang B., Li K., Zhong X., Zou J. (2022). Implementation of deep learning in drug design. MedComm-Future Med..

[38] Cai C., Wang S., Xu Y., Zhang W., Tang K., Ouyang Q., Lai L., Pei J. (2020). Transfer Learning for Drug Discovery. J. Med. Chem..

[39] Liu F., Dai Y. (2023). Product quality prediction method in small sample data environment. Adv. Eng. Inform..

[40] Fan F.J., Shi Y. (2022). Effects of data quality and quantity on deep learning for protein-ligand binding affinity prediction. Bioorg. Med. Chem..

[41] Peters M.E., Neumann M., Iyyer M., Gardner G., Clark C., Lee K., Zettlemoyer L. (2018). Deep contextualized word representations. Proceedings of the 2018 Conference of the North American Chapter of the Association for Computational Linguistics: Human Language Technologies.

[42] Radford A., Wu J., Child R., Luan D., Amodei D., Sutskever I. (2019). Language models are unsupervised multitask learners. OpenAI Blog.

[43] Devlin J., Chang M.W., Lee K., Toutanova K. (2019). BERT: Pre-training of Deep Bidirectional Transformers for Language Understanding. Proceedings of the 2019 Conference of the North American Chapter of the Association for Computational Linguistics: Human Language Technologies.

[44] Apweiler R., Bairoch A., Wu C.H., Barker W.C., Boeckmann B., Ferro S., Gasteiger E., Huang H., Lopez R., Magrane M. (2004). UniProt: The Universal Protein knowledgebase. Nucleic Acids Res..

[45] Bepler T., Berger B. (2019). Learning Protein Sequence Embeddings Using Information from Structure. https://openreview.net/forum?id=SygLehCqtm.

[46] Min S., Park S., Kim S., Choi H.S., Lee B., Yoon S. (2021). Pre-Training of Deep Bidirectional Protein Sequence Representations With Structural Information. IEEE Access.

[47] Heinzinger M., Elnaggar A., Wang Y., Dallago C., Nechaev D., Matthes F., Rost. B. (2019). Modeling aspects of the language of life through transfer-learning protein sequences. BMC Bioinform..

[48] Rives A., Meier J., Sercu T., Goyal S., Lin Z., Liu J., Guo D., Ott M., Zitnick C.L., Ma J. (2021). Biological structure and function emerge from scaling unsupervised learning to 250 million protein sequences. Proc. Natl. Acad. Sci. USA.

[49] Elnaggar A., Heinzinger M., Dallago C., Rehawi G., Wang Y., Jones L., Gibbs T., Feher T., Angerer C., Steinegger M. (2022). ProtTrans: Toward Understanding the Language of Life Through Self-Supervised Learning. IEEE Trans. Pattern Anal. Mach. Intell..

[50] Stärk H., Dallago C., Heinzinger M., Rost B. (2021). Light attention predicts protein location from the language of life. Bioinform. Adv..

[51] Salem M., Arshadi A.K., Yuan J.S. (2021). AMPDeep: Hemolytic activity prediction of antimicrobial peptides using transfer learning. BMC Bioinform..

[52] Jiang J., Lin X., Jiang Y., Jiang L., Lv Z. (2022). Identify Bitter Peptides by Using Deep Representation Learning Features. Int. J. Mol. Sci..

[53] Zhang J., Yang L., Tian Z., Zhao W., Sun C., Zhu L., Huang M., Guo G., Liang G. (2022). Large-Scale Screening of Antifungal Peptides Based on Quantitative Structure—Activity Relationship. ACS Med. Chem. Lett..

[54] Sharma R., Shrivastava S., Singh S.K., Kumar A., Saxena S., Singh R.K. (2022). Deep-AFPpred: Identifying novel antifungal peptides using pretrained embeddings from seq2vec with 1DCNN-BiLSTM. Brief. Bioinform..

[55] Li W., Godzik A. (2006). Cd-hit: A fast program for clustering and comparing large sets of protein or nucleotide sequences. Bioinformatics.

[56] Chicco D., Jurman G. (2020). The advantages of the Matthews correlation coefficient (MCC) over F1 score and accuracy in binary classification evaluation. BMC Genom..

[57] Agrawal P., Bhalla S., Chaudhary K., Kumar R., Sharma M., Raghava G.P.S. (2018). In Silico Approach for Prediction of Antifungal Peptides. Front. Microbiol..

[58] Dallago C., Schütze K., Heinzinger M., Olenyi T., Littmann M., Lu A.X., Yang K.K., Min S., Yoon S., Morton J.T. (2021). Learned embeddings from deep learning to visualize and predict protein sets. Curr. Protoc..

[59] Peptides—PyPI. https://pypi.org/project/peptides/.

[60] Pedregosa F., Varoquaux G., Gramfort A., Michel V., Thirion B., Grisel O., Blondel M., Prettenhofer P., Weiss R., Dubourg V. (2011). Scikit-learn: Machine Learning in Python. J. Mach. Learn. Res..

[61] Antifungal Webserver. https://www.chemoinfolab.com/antifungal/.

[62] Deep-AFPpred. https://afppred.anvil.app/.

[63] AntiFP. https://webs.iiitd.edu.in/raghava/antifp/.

