# Supplementary material for: Prediction of Antifungal Activity of Antimicrobial Peptides by Transfer Learning from Protein Pretrained Models
Source: Int J Mol Sci. 2023 Jun 17;24(12):10270. doi: 10.3390/ijms241210270 (PMC10299371; doi:10.3390/ijms241210270)
Supplement: Supplementary file 1 [file ijms-24-10270-s001.zip › ijms-2434315-supplementary.pdf]

Prediction of antifungal activity in antimicrobial peptides employing transfer learning from protein pretrained models.

Fernando Lobo, Maily González, Alicia Boto, José Manuel Pérez de la Lastra

SUPPORTING INFORMATION

Page

2 **Table S1:** Parameters Grid:

3 **Table S2:** Best parameters found by Grid Cross-Validation

10 **Table S3:** Models metrics

45 **Table S4:** Models metrics averaging over all feature-selector methods

TABLE S1

Parameters Grid

Classifier	Parameters	Values
SVC	C<br>kernel	0.1-1000 (log scale)<br>rfb or poly
Logistic Regression	C<br>solver	0.1-100 (log scale)<br>sag or saga
Multi-Layer Perceptron	activation<br>alpha<br>hidden_layers_sizes	logistic, tanh or relu<br>0.2, 0.1, 0.05 or 0.01<br>(10,) - (30,) / (5,5) – (15,5)
k-Nearest Neighbors	n_neighbors<br>weights<br>metric	5-15<br>uniform or distance<br>l1 or l2
Random Forest	n_estimators<br>criterion<br>max_depth	30-150<br>gini or entropy<br>2-15

TABLE S2

Best Parameters found by Grid Cross Validation

ProtT5

	pca	no	rfe	kb	sfm
LR_logC	-0.690 ± 0.223	-0.524 ± 0.347	-0.090 ± 0.619	0.283 ± 0.267	0.055 ± 0.279
LR_solver	sag	sag	sag	sag	sag
MLP_activation	logistic	logistic	logistic	logistic	logistic
MLP_alpha	0.200 ± 0.000	0.160 ± 0.068	0.200 ± 0.000	0.130 ± 0.083	0.180 ± 0.056
MLP_hidden_layer_sizes	(10,)	(30,)	[(15,) (25,)]	(25,)	(25,)
RF_criterion	entropy	entropy	entropy	entropy	entropy
RF_max_depth	4.800 ± 1.362	9.600 ± 1.417	10.200 ± 3.666	10.400 ± 1.668	11.200 ± 2.224
RF_n_estimators	132.000 ± 16.207	76.000 ± 40.849	88.000 ± 28.345	116.000 ± 39.892	110.000 ± 39.307
SVC_kernel	rbf	rbf	rbf	rbf	rbf
SVC_logC	0.021 ± 0.376	0.131 ± 0.254	0.214 ± 0.372	0.297 ± 0.195	0.048 ± 0.463
kNN_metric	l2	l2	l2	l1	l2
kNN_n_neighbors	12.600 ± 3.241	9.800 ± 3.770	12.600 ± 4.085	13.400 ± 2.723	10.200 ± 3.770
kNN_weights	distance	distance	distance	distance	uniform

SeqVec

	pca	no	rfe	kb	sfm
LR_logC	0.572 ± 0.493	0.614 ± 0.370	0.614 ± 0.347	0.469 ± 0.168	0.531 ± 0.357
LR_solver	sag	sag	sag	sag	sag
MLP_activation	relu	relu	relu	relu	relu
MLP_alpha	0.130 ± 0.083	0.120 ± 0.094	0.080 ± 0.034	0.150 ± 0.088	0.130 ± 0.083
MLP_hidden_layer_sizes	(35,)	[(30,) (35,)]	(35,)	[(15, 5) (35,)]	(30,)
RF_criterion	entropy	entropy	entropy	entropy	entropy
RF_max_depth	11.000 ± 2.325	12.800 ± 0.556	12.800 ± 1.040	12.800 ± 0.556	11.800 ± 1.362
RF_n_estimators	120.000 ± 26.368	110.000 ± 42.152	112.000 ± 37.702	122.000 ± 22.235	86.000 ± 16.677
SVC_kernel	rbf	rbf	rbf	rbf	rbf
SVC_logC	0.903 ± 0.307	0.986 ± 0.334	1.069 ± 0.171	1.179 ± 0.372	0.931 ± 0.454
kNN_metric	l2	l1	l1	l2	l1
kNN_n_neighbors	6.600 ± 1.112	6.600 ± 1.112	6.600 ± 2.080	5.000 ± 0.000	5.800 ± 2.224
kNN_weights	distance	distance	distance	distance	distance

ESM1b

	pca	no	rfe	kb	sfm
LR_logC	0.469 ± 0.357	0.966 ± 0.352	0.759 ± 0.375	0.759 ± 0.288	0.779 ± 0.357
LR_solver	sag	saga	sag	sag	saga
MLP_activation	relu	relu	relu	relu	relu
MLP_alpha	0.120 ± 0.094	0.056 ± 0.102	0.120 ± 0.094	0.160 ± 0.068	0.072 ± 0.051
MLP_hidden_layer_sizes	(35,)	(35,)	[(30,) (35,)]	(15,)	(35,)
RF_criterion	entropy	entropy	entropy	entropy	entropy
RF_max_depth	12.400 ± 1.112	11.800 ± 1.362	12.000 ± 1.758	12.000 ± 1.522	12.800 ± 1.040
RF_n_estimators	120.000 ± 36.239	114.000 ± 16.677	88.000 ± 20.425	110.000 ± 31.690	88.000 ± 35.594
SVC_kernel	rbf	rbf	rbf	rbf	poly
SVC_logC	0.766 ± 0.282	2.200 ± 0.548	1.483 ± 0.822	1.014 ± 0.311	1.400 ± 1.068
kNN_metric	l2	l1	l1	l1	l1
kNN_n_neighbors	6.600 ± 1.112	7.000 ± 0.000	6.600 ± 2.080	7.000 ± 2.486	7.400 ± 2.723
kNN_weights	distance	distance	distance	distance	distance

Bepler

	pca	no	rfe	kb	sfm
LR_logC	1.462 ± 0.671	1.834 ± 0.070	1.607 ± 0.533	1.483 ± 0.643	1.607 ± 0.556
LR_solver	sag	sag	sag	sag	sag
MLP_activation	tanh	tanh	logistic	tanh	tanh
MLP_alpha	0.010 ± 0.000	0.010 ± 0.000	0.028 ± 0.050	0.010 ± 0.000	0.034 ± 0.027
MLP_hidden_layer_sizes	(35,)	[(10,) (10, 5) (15,) (25,) (30,)]	(35,)	[(15,) (35,)]	(10,)
RF_criterion	entropy	gini	entropy	entropy	entropy
RF_max_depth	12.200 ± 1.844	13.600 ± 1.112	11.200 ± 2.042	12.600 ± 1.417	13.000 ± 1.522
RF_n_estimators	124.000 ± 20.799	112.000 ± 22.235	128.000 ± 5.559	132.000 ± 10.400	124.000 ± 20.799
SVC_kernel	rbf	rbf	rbf	rbf	rbf
SVC_logC	1.262 ± 0.753	2.145 ± 0.143	1.869 ± 0.460	2.338 ± 0.330	2.117 ± 0.230
kNN_metric	l1	l1	l1	l1	l1
kNN_n_neighbors	7.000 ± 3.516	6.600 ± 1.112	8.200 ± 2.224	6.600 ± 2.080	7.000 ± 2.486
kNN_weights	distance	distance	distance	distance	distance

plusRNN

	pca	no	rfe	kb	sfm
LR_logC	1.566 ± 0.379	1.503 ± 0.677	1.648 ± 0.195	1.503 ± 0.168	1.483 ± 0.643
LR_solver	sag	sag	sag	sag	sag
MLP_activation	relu	relu	relu	relu	relu
MLP_alpha	0.110 ± 0.068	0.090 ± 0.081	0.064 ± 0.098	0.112 ± 0.107	0.034 ± 0.027
MLP_hidden_layer_sizes	(35,)	[(15,) (35,)]	(35,)	[(15,) (15, 5)]	(20,)
RF_criterion	entropy	entropy	entropy	entropy	gini
RF_max_depth	8.400 ± 2.994	12.600 ± 1.885	11.400 ± 2.723	12.400 ± 1.112	12.400 ± 1.112
RF_n_estimators	132.000 ± 16.207	114.000 ± 46.175	108.000 ± 47.657	110.000 ± 26.368	112.000 ± 23.910
SVC_kernel	rbf	rbf	rbf	rbf	rbf
SVC_logC	0.710 ± 0.430	0.931 ± 0.528	0.876 ± 0.494	0.766 ± 0.427	0.903 ± 0.254
kNN_metric	l2	l1	l1	l2	l1
kNN_n_neighbors	5.400 ± 1.112	7.800 ± 4.846	9.400 ± 2.723	10.200 ± 2.224	7.800 ± 2.834
kNN_weights	distance	distance	distance	distance	distance

Descriptors

	pca	no	rfe	kb	sfm
LR_logC	1.710 ± 0.279	1.628 ± 0.586	1.359 ± 0.815	1.276 ± 0.716	1.276 ± 0.636
LR_solver	sag	sag	sag	sag	sag
MLP_activation	tanh	tanh	tanh	['logistic' 'relu']	tanh
MLP_alpha	0.142 ± 0.106	0.104 ± 0.118	0.124 ± 0.129	0.062 ± 0.048	0.102 ± 0.113
MLP_hidden_layer_sizes	[(20,) (30,)]	(35,)	(25,)	(30,)	[(30,) (35,)]
RF_criterion	entropy	entropy	entropy	entropy	entropy
RF_max_depth	13.200 ± 1.621	12.800 ± 1.362	12.600 ± 1.417	13.200 ± 1.040	12.600 ± 2.080
RF_n_estimators	118.000 ± 30.950	106.000 ± 38.912	88.000 ± 38.713	106.000 ± 25.775	108.000 ± 22.235
SVC_kernel	rbf	rbf	rbf	rbf	rbf
SVC_logC	0.848 ± 0.311	1.703 ± 0.195	1.924 ± 0.843	1.869 ± 0.224	1.731 ± 0.307
kNN_metric	l1	l1	l1	l1	l1
kNN_n_neighbors	8.200 ± 3.770	7.000 ± 3.045	8.200 ± 4.516	7.400 ± 2.723	6.200 ± 1.362
kNN_weights	distance	distance	distance	distance	distance

Prottrans

	pca	no	rfe	kb	sfm
LR_logC	0.821 ± 0.511	0.717 ± 0.215	0.490 ± 0.614	0.945 ± 0.279	0.903 ± 0.535
LR_solver	sag	sag	sag	sag	sag
MLP_activation	relu	relu	relu	relu	relu
MLP_alpha	0.110 ± 0.068	0.160 ± 0.068	0.162 ± 0.106	0.150 ± 0.088	0.142 ± 0.106
MLP_hidden_layer_sizes	(25,)	[(30,) (35,)]	(25,)	(35,)	(25,)
RF_criterion	gini	entropy	gini	gini	gini
RF_max_depth	11.600 ± 2.080	11.000 ± 1.965	13.000 ± 2.153	13.000 ± 2.153	12.600 ± 1.417
RF_n_estimators	126.000 ± 14.172	104.000 ± 43.594	96.000 ± 35.810	106.000 ± 22.580	94.000 ± 39.892
SVC_kernel	rbf	rbf	rbf	rbf	rbf
SVC_logC	0.490 ± 0.143	0.793 ± 0.686	0.572 ± 0.094	0.876 ± 0.463	0.462 ± 0.413
kNN_metric	l2	l1	l1	l1	l1
kNN_n_neighbors	9.000 ± 4.651	8.600 ± 6.190	9.400 ± 4.782	7.000 ± 4.306	5.800 ± 2.224
kNN_weights	distance	distance	distance	distance	distance

TABLE S3
Models Metrics

Descriptors

kb

	Train Dataset					Test Dataset				
	Acc.	Prec.	Recall	F1	MCC	Acc.	Prec.	Recall	F1	MCC
SVC	0.926 ± 0.007	0.927 ± 0.006	0.924 ± 0.009	0.925 ± 0.007	0.851 ± 0.015	0.872 ± 0.010	0.856 ± 0.011	0.894 ± 0.009	0.875 ± 0.010	0.745 ± 0.021
LR	0.815 ± 0.004	0.807 ± 0.001	0.828 ± 0.008	0.817 ± 0.004	0.630 ± 0.007	0.843 ± 0.002	0.848 ± 0.004	0.836 ± 0.010	0.842 ± 0.003	0.686 ± 0.003
MLP	0.922 ± 0.020	0.921 ± 0.020	0.922 ± 0.021	0.922 ± 0.020	0.843 ± 0.040	0.855 ± 0.015	0.843 ± 0.010	0.874 ± 0.031	0.858 ± 0.017	0.711 ± 0.030
kNN	1.000 ± 0.000	1.000 ± 0.000	1.000 ± 0.000	1.000 ± 0.000	1.000 ± 0.000	0.845 ± 0.005	0.822 ± 0.007	0.880 ± 0.003	0.850 ± 0.005	0.691 ± 0.010
RF	1.000 ± 0.000	1.000 ± 0.000	1.000 ± 0.001	1.000 ± 0.000	1.000 ± 0.001	0.877 ± 0.004	0.871 ± 0.004	0.885 ± 0.007	0.878 ± 0.004	0.754 ± 0.008
Stack.	0.989 ± 0.005	0.992 ± 0.002	0.986 ± 0.007	0.989 ± 0.005	0.978 ± 0.009	0.879 ± 0.008	0.870 ± 0.010	0.891 ± 0.012	0.880 ± 0.008	0.758 ± 0.017

pca

	Train Dataset					Test Dataset				
	Acc.	Prec.	Recall	F1	MCC	Acc.	Prec.	Recall	F1	MCC
SVC	0.934 ± 0.012	0.934 ± 0.010	0.934 ± 0.015	0.934 ± 0.012	0.867 ± 0.025	0.882 ± 0.003	0.865 ± 0.002	0.904 ± 0.005	0.884 ± 0.003	0.764 ± 0.007
LR	0.817 ± 0.000	0.807 ± 0.001	0.833 ± 0.000	0.820 ± 0.000	0.635 ± 0.001	0.847 ± 0.000	0.852 ± 0.000	0.840 ± 0.000	0.846 ± 0.000	0.693 ± 0.000
MLP	0.921 ± 0.007	0.919 ± 0.008	0.924 ± 0.006	0.921 ± 0.007	0.842 ± 0.014	0.857 ± 0.012	0.846 ± 0.005	0.874 ± 0.025	0.859 ± 0.014	0.715 ± 0.025
kNN	1.000 ± 0.000	1.000 ± 0.000	1.000 ± 0.000	1.000 ± 0.000	1.000 ± 0.000	0.846 ± 0.010	0.818 ± 0.013	0.892 ± 0.007	0.853 ± 0.008	0.695 ± 0.019
RF	0.998 ± 0.005	0.997 ± 0.007	0.999 ± 0.003	0.998 ± 0.005	0.996 ± 0.010	0.860 ± 0.006	0.854 ± 0.008	0.868 ± 0.012	0.861 ± 0.006	0.720 ± 0.012
Stack.	0.987 ± 0.005	0.990 ± 0.005	0.984 ± 0.005	0.987 ± 0.005	0.974 ± 0.010	0.875 ± 0.007	0.867 ± 0.008	0.887 ± 0.009	0.877 ± 0.007	0.751 ± 0.014

sfm

	Train Dataset					Test Dataset				
	Acc.	Prec.	Recall	F1	MCC	Acc.	Prec.	Recall	F1	MCC
SVC	0.923 ± 0.009	0.925 ± 0.005	0.921 ± 0.014	0.923 ± 0.009	0.846 ± 0.018	0.876 ± 0.009	0.861 ± 0.010	0.897 ± 0.008	0.879 ± 0.009	0.753 ± 0.018
LR	0.815 ± 0.003	0.807 ± 0.002	0.829 ± 0.006	0.818 ± 0.004	0.631 ± 0.007	0.842 ± 0.002	0.848 ± 0.007	0.834 ± 0.008	0.841 ± 0.002	0.684 ± 0.004
MLP	0.938 ± 0.027	0.934 ± 0.029	0.941 ± 0.026	0.938 ± 0.027	0.875 ± 0.054	0.860 ± 0.022	0.847 ± 0.023	0.879 ± 0.024	0.863 ± 0.022	0.721 ± 0.045
kNN	1.000 ± 0.000	1.000 ± 0.000	1.000 ± 0.000	1.000 ± 0.000	1.000 ± 0.000	0.847 ± 0.000	0.825 ± 0.002	0.880 ± 0.003	0.852 ± 0.000	0.695 ± 0.000
RF	0.998 ± 0.005	0.999 ± 0.003	0.998 ± 0.007	0.998 ± 0.005	0.997 ± 0.009	0.871 ± 0.003	0.865 ± 0.006	0.880 ± 0.007	0.872 ± 0.003	0.743 ± 0.007
Stack.	0.991 ± 0.004	0.995 ± 0.002	0.988 ± 0.005	0.991 ± 0.004	0.982 ± 0.007	0.882 ± 0.007	0.874 ± 0.006	0.892 ± 0.010	0.883 ± 0.007	0.763 ± 0.013

rfe

	Train Dataset					Test Dataset				
	Acc.	Prec.	Recall	F1	MCC	Acc.	Prec.	Recall	F1	MCC
SVC	0.929 ± 0.023	0.929 ± 0.021	0.930 ± 0.026	0.929 ± 0.023	0.858 ± 0.046	0.868 ± 0.007	0.853 ± 0.013	0.890 ± 0.009	0.871 ± 0.006	0.737 ± 0.013
LR	0.815 ± 0.003	0.807 ± 0.002	0.827 ± 0.006	0.817 ± 0.003	0.630 ± 0.005	0.843 ± 0.002	0.849 ± 0.005	0.834 ± 0.006	0.842 ± 0.002	0.686 ± 0.003
MLP	0.918 ± 0.008	0.916 ± 0.012	0.919 ± 0.012	0.918 ± 0.008	0.836 ± 0.016	0.867 ± 0.009	0.856 ± 0.017	0.882 ± 0.013	0.869 ± 0.008	0.734 ± 0.017
kNN	1.000 ± 0.000	1.000 ± 0.000	1.000 ± 0.000	1.000 ± 0.000	1.000 ± 0.000	0.843 ± 0.011	0.821 ± 0.010	0.877 ± 0.013	0.848 ± 0.011	0.688 ± 0.022
RF	0.999 ± 0.001	0.999 ± 0.001	1.000 ± 0.001	0.999 ± 0.001	0.999 ± 0.003	0.872 ± 0.008	0.859 ± 0.014	0.891 ± 0.013	0.874 ± 0.008	0.745 ± 0.016
Stack.	0.988 ± 0.010	0.991 ± 0.008	0.984 ± 0.013	0.988 ± 0.010	0.976 ± 0.020	0.882 ± 0.006	0.872 ± 0.006	0.894 ± 0.007	0.883 ± 0.006	0.763 ± 0.011

no

	Train Dataset					Test Dataset				
	Acc.	Prec.	Recall	F1	MCC	Acc.	Prec.	Recall	F1	MCC
SVC	0.918 ± 0.007	0.921 ± 0.006	0.915 ± 0.009	0.918 ± 0.008	0.837 ± 0.015	0.877 ± 0.007	0.863 ± 0.005	0.896 ± 0.010	0.879 ± 0.007	0.754 ± 0.014
LR	0.817 ± 0.002	0.808 ± 0.002	0.831 ± 0.003	0.819 ± 0.002	0.634 ± 0.004	0.842 ± 0.002	0.850 ± 0.005	0.831 ± 0.003	0.840 ± 0.001	0.684 ± 0.004
MLP	0.929 ± 0.017	0.924 ± 0.017	0.935 ± 0.019	0.930 ± 0.017	0.859 ± 0.034	0.862 ± 0.008	0.847 ± 0.016	0.884 ± 0.005	0.865 ± 0.006	0.724 ± 0.015
kNN	1.000 ± 0.000	1.000 ± 0.000	1.000 ± 0.000	1.000 ± 0.000	1.000 ± 0.000	0.845 ± 0.005	0.823 ± 0.007	0.879 ± 0.003	0.850 ± 0.005	0.691 ± 0.010
RF	1.000 ± 0.000	1.000 ± 0.000	1.000 ± 0.000	1.000 ± 0.000	1.000 ± 0.000	0.868 ± 0.009	0.864 ± 0.009	0.875 ± 0.013	0.869 ± 0.009	0.737 ± 0.018
Stack.	0.989 ± 0.006	0.991 ± 0.005	0.987 ± 0.007	0.989 ± 0.006	0.978 ± 0.011	0.881 ± 0.004	0.872 ± 0.006	0.892 ± 0.010	0.882 ± 0.005	0.762 ± 0.009

SeqVec

kb

	Train Dataset					Test Dataset				
	Acc.	Prec.	Recall	F1	MCC	Acc.	Prec.	Recall	F1	MCC
SVC	0.999 ± 0.001	0.999 ± 0.001	1.000 ± 0.000	0.999 ± 0.001	0.999 ± 0.001	0.910 ± 0.009	0.923 ± 0.022	0.895 ± 0.006	0.909 ± 0.008	0.820 ± 0.018
LR	0.938 ± 0.005	0.937 ± 0.008	0.939 ± 0.001	0.938 ± 0.005	0.876 ± 0.010	0.893 ± 0.002	0.906 ± 0.005	0.877 ± 0.000	0.892 ± 0.002	0.787 ± 0.005
MLP	0.997 ± 0.008	0.996 ± 0.010	0.997 ± 0.006	0.997 ± 0.008	0.993 ± 0.015	0.890 ± 0.010	0.896 ± 0.027	0.884 ± 0.016	0.889 ± 0.008	0.781 ± 0.020
kNN	1.000 ± 0.000	1.000 ± 0.000	1.000 ± 0.000	1.000 ± 0.000	1.000 ± 0.000	0.903 ± 0.008	0.891 ± 0.014	0.920 ± 0.000	0.905 ± 0.007	0.807 ± 0.016
RF	1.000 ± 0.000	1.000 ± 0.000	1.000 ± 0.000	1.000 ± 0.000	1.000 ± 0.000	0.898 ± 0.010	0.915 ± 0.009	0.876 ± 0.017	0.895 ± 0.011	0.796 ± 0.021
Stack.	1.000 ± 0.000	1.000 ± 0.000	1.000 ± 0.000	1.000 ± 0.000	1.000 ± 0.000	0.923 ± 0.004	0.942 ± 0.011	0.901 ± 0.007	0.921 ± 0.004	0.846 ± 0.008

pca

	Train Dataset					Test Dataset				
	Acc.	Prec.	Recall	F1	MCC	Acc.	Prec.	Recall	F1	MCC
SVC	0.999 ± 0.002	0.999 ± 0.002	0.999 ± 0.002	0.999 ± 0.002	0.998 ± 0.003	0.919 ± 0.003	0.923 ± 0.003	0.915 ± 0.004	0.919 ± 0.003	0.839 ± 0.006
LR	0.971 ± 0.013	0.969 ± 0.014	0.973 ± 0.013	0.971 ± 0.013	0.943 ± 0.027	0.879 ± 0.011	0.891 ± 0.009	0.863 ± 0.017	0.877 ± 0.012	0.758 ± 0.022
MLP	1.000 ± 0.000	1.000 ± 0.000	1.000 ± 0.000	1.000 ± 0.000	1.000 ± 0.000	0.892 ± 0.004	0.903 ± 0.009	0.877 ± 0.009	0.890 ± 0.004	0.783 ± 0.008
kNN	1.000 ± 0.000	1.000 ± 0.000	1.000 ± 0.000	1.000 ± 0.000	1.000 ± 0.000	0.892 ± 0.003	0.867 ± 0.001	0.926 ± 0.005	0.896 ± 0.003	0.787 ± 0.006
RF	0.999 ± 0.004	0.999 ± 0.003	0.999 ± 0.004	0.999 ± 0.004	0.997 ± 0.007	0.878 ± 0.016	0.884 ± 0.020	0.871 ± 0.017	0.877 ± 0.016	0.757 ± 0.033
Stack.	1.000 ± 0.000	1.000 ± 0.000	1.000 ± 0.000	1.000 ± 0.000	1.000 ± 0.000	0.908 ± 0.007	0.915 ± 0.005	0.900 ± 0.013	0.907 ± 0.008	0.816 ± 0.015

sfm

	Train Dataset					Test Dataset				
	Acc.	Prec.	Recall	F1	MCC	Acc.	Prec.	Recall	F1	MCC
SVC	0.992 ± 0.016	0.993 ± 0.015	0.991 ± 0.018	0.992 ± 0.016	0.984 ± 0.033	0.904 ± 0.012	0.911 ± 0.015	0.895 ± 0.010	0.903 ± 0.011	0.808 ± 0.023
LR	0.927 ± 0.009	0.925 ± 0.009	0.929 ± 0.011	0.927 ± 0.009	0.854 ± 0.019	0.892 ± 0.007	0.901 ± 0.005	0.881 ± 0.010	0.891 ± 0.007	0.784 ± 0.013
MLP	0.987 ± 0.013	0.989 ± 0.012	0.984 ± 0.015	0.987 ± 0.013	0.974 ± 0.026	0.897 ± 0.007	0.905 ± 0.009	0.888 ± 0.017	0.896 ± 0.008	0.795 ± 0.013
kNN	1.000 ± 0.000	1.000 ± 0.000	1.000 ± 0.000	1.000 ± 0.000	1.000 ± 0.000	0.897 ± 0.008	0.873 ± 0.008	0.929 ± 0.012	0.900 ± 0.008	0.796 ± 0.016
RF	1.000 ± 0.000	1.000 ± 0.000	1.000 ± 0.000	1.000 ± 0.000	1.000 ± 0.000	0.895 ± 0.005	0.908 ± 0.006	0.879 ± 0.011	0.893 ± 0.006	0.790 ± 0.010
Stack.	0.999 ± 0.003	1.000 ± 0.001	0.999 ± 0.004	0.999 ± 0.003	0.998 ± 0.005	0.919 ± 0.013	0.927 ± 0.018	0.909 ± 0.010	0.918 ± 0.013	0.838 ± 0.027

rfe

	Train Dataset					Test Dataset				
	Acc.	Prec.	Recall	F1	MCC	Acc.	Prec.	Recall	F1	MCC
SVC	0.999 ± 0.002	0.999 ± 0.001	0.999 ± 0.002	0.999 ± 0.002	0.998 ± 0.003	0.913 ± 0.006	0.921 ± 0.008	0.904 ± 0.012	0.912 ± 0.006	0.827 ± 0.011
LR	0.971 ± 0.019	0.970 ± 0.019	0.972 ± 0.019	0.971 ± 0.019	0.942 ± 0.038	0.886 ± 0.010	0.889 ± 0.017	0.883 ± 0.006	0.886 ± 0.009	0.773 ± 0.019
MLP	1.000 ± 0.000	1.000 ± 0.000	1.000 ± 0.001	1.000 ± 0.000	1.000 ± 0.001	0.892 ± 0.009	0.896 ± 0.012	0.886 ± 0.009	0.891 ± 0.009	0.783 ± 0.018
kNN	1.000 ± 0.000	1.000 ± 0.000	1.000 ± 0.000	1.000 ± 0.000	1.000 ± 0.000	0.897 ± 0.009	0.872 ± 0.003	0.930 ± 0.019	0.900 ± 0.010	0.795 ± 0.019
RF	1.000 ± 0.000	1.000 ± 0.000	1.000 ± 0.000	1.000 ± 0.000	1.000 ± 0.000	0.899 ± 0.017	0.909 ± 0.020	0.887 ± 0.014	0.898 ± 0.017	0.798 ± 0.033
Stack.	1.000 ± 0.000	1.000 ± 0.000	1.000 ± 0.000	1.000 ± 0.000	1.000 ± 0.000	0.922 ± 0.006	0.932 ± 0.008	0.911 ± 0.005	0.921 ± 0.006	0.845 ± 0.012

no

	Train Dataset					Test Dataset				
	Acc.	Prec.	Recall	F1	MCC	Acc.	Prec.	Recall	F1	MCC
SVC	0.997 ± 0.005	0.998 ± 0.004	0.997 ± 0.006	0.997 ± 0.005	0.994 ± 0.011	0.916 ± 0.008	0.924 ± 0.010	0.906 ± 0.006	0.915 ± 0.008	0.831 ± 0.015
LR	0.981 ± 0.012	0.980 ± 0.013	0.982 ± 0.012	0.981 ± 0.012	0.962 ± 0.025	0.880 ± 0.007	0.888 ± 0.011	0.871 ± 0.003	0.879 ± 0.007	0.761 ± 0.015
MLP	1.000 ± 0.000	1.000 ± 0.001	1.000 ± 0.000	1.000 ± 0.000	1.000 ± 0.001	0.884 ± 0.008	0.887 ± 0.016	0.880 ± 0.015	0.884 ± 0.007	0.768 ± 0.015
kNN	1.000 ± 0.000	1.000 ± 0.000	1.000 ± 0.000	1.000 ± 0.000	1.000 ± 0.000	0.899 ± 0.000	0.871 ± 0.004	0.936 ± 0.005	0.902 ± 0.000	0.799 ± 0.001
RF	1.000 ± 0.000	1.000 ± 0.000	1.000 ± 0.000	1.000 ± 0.000	1.000 ± 0.000	0.899 ± 0.004	0.910 ± 0.011	0.886 ± 0.006	0.898 ± 0.003	0.798 ± 0.008
Stack.	1.000 ± 0.001	1.000 ± 0.000	0.999 ± 0.002	1.000 ± 0.001	0.999 ± 0.002	0.917 ± 0.008	0.925 ± 0.012	0.907 ± 0.008	0.916 ± 0.008	0.833 ± 0.016

ESM1b

kb

	Train Dataset					Test Dataset				
	Acc.	Prec.	Recall	F1	MCC	Acc.	Prec.	Recall	F1	MCC
SVC	0.982 ± 0.013	0.986 ± 0.012	0.978 ± 0.015	0.982 ± 0.013	0.965 ± 0.026	0.886 ± 0.009	0.889 ± 0.008	0.883 ± 0.010	0.886 ± 0.009	0.773 ± 0.018
LR	0.935 ± 0.009	0.935 ± 0.010	0.935 ± 0.007	0.935 ± 0.009	0.871 ± 0.017	0.871 ± 0.006	0.875 ± 0.006	0.865 ± 0.005	0.870 ± 0.006	0.742 ± 0.011
MLP	0.959 ± 0.025	0.953 ± 0.029	0.967 ± 0.021	0.960 ± 0.025	0.919 ± 0.050	0.871 ± 0.003	0.863 ± 0.008	0.883 ± 0.011	0.873 ± 0.004	0.743 ± 0.007
kNN	1.000 ± 0.000	1.000 ± 0.000	1.000 ± 0.000	1.000 ± 0.000	1.000 ± 0.000	0.878 ± 0.003	0.884 ± 0.008	0.870 ± 0.014	0.877 ± 0.004	0.756 ± 0.006
RF	1.000 ± 0.000	1.000 ± 0.000	1.000 ± 0.001	1.000 ± 0.000	1.000 ± 0.001	0.883 ± 0.007	0.901 ± 0.007	0.862 ± 0.013	0.881 ± 0.007	0.768 ± 0.013
Stack.	0.993 ± 0.003	0.995 ± 0.003	0.990 ± 0.004	0.993 ± 0.003	0.985 ± 0.007	0.889 ± 0.005	0.894 ± 0.006	0.882 ± 0.006	0.888 ± 0.005	0.777 ± 0.010

pca

	Train Dataset					Test Dataset				
	Acc.	Prec.	Recall	F1	MCC	Acc.	Prec.	Recall	F1	MCC
SVC	0.983 ± 0.014	0.985 ± 0.013	0.981 ± 0.016	0.983 ± 0.014	0.966 ± 0.028	0.887 ± 0.008	0.885 ± 0.016	0.891 ± 0.003	0.888 ± 0.007	0.775 ± 0.016
LR	0.961 ± 0.008	0.960 ± 0.012	0.961 ± 0.004	0.961 ± 0.008	0.922 ± 0.017	0.879 ± 0.004	0.886 ± 0.003	0.869 ± 0.008	0.878 ± 0.005	0.758 ± 0.009
MLP	0.998 ± 0.003	1.000 ± 0.001	0.997 ± 0.004	0.998 ± 0.003	0.997 ± 0.005	0.876 ± 0.010	0.884 ± 0.013	0.867 ± 0.017	0.875 ± 0.011	0.753 ± 0.021
kNN	1.000 ± 0.000	1.000 ± 0.000	1.000 ± 0.000	1.000 ± 0.000	1.000 ± 0.000	0.882 ± 0.001	0.893 ± 0.011	0.867 ± 0.010	0.880 ± 0.000	0.764 ± 0.003
RF	1.000 ± 0.000	1.000 ± 0.000	1.000 ± 0.000	1.000 ± 0.000	1.000 ± 0.000	0.889 ± 0.013	0.897 ± 0.015	0.879 ± 0.021	0.888 ± 0.014	0.779 ± 0.026
Stack.	0.993 ± 0.002	0.995 ± 0.004	0.992 ± 0.001	0.993 ± 0.002	0.987 ± 0.005	0.889 ± 0.005	0.889 ± 0.007	0.890 ± 0.010	0.889 ± 0.005	0.778 ± 0.009

sfm

	Train Dataset					Test Dataset				
	Acc.	Prec.	Recall	F1	MCC	Acc.	Prec.	Recall	F1	MCC
SVC	0.974 ± 0.028	0.977 ± 0.030	0.970 ± 0.027	0.974 ± 0.028	0.948 ± 0.057	0.878 ± 0.017	0.882 ± 0.020	0.873 ± 0.020	0.877 ± 0.017	0.756 ± 0.034
LR	0.919 ± 0.006	0.920 ± 0.007	0.917 ± 0.006	0.919 ± 0.006	0.838 ± 0.012	0.872 ± 0.019	0.884 ± 0.030	0.858 ± 0.014	0.870 ± 0.018	0.745 ± 0.039
MLP	0.989 ± 0.005	0.990 ± 0.004	0.987 ± 0.008	0.989 ± 0.005	0.978 ± 0.010	0.870 ± 0.016	0.873 ± 0.020	0.867 ± 0.021	0.870 ± 0.016	0.741 ± 0.032
kNN	1.000 ± 0.000	1.000 ± 0.000	1.000 ± 0.000	1.000 ± 0.000	1.000 ± 0.000	0.882 ± 0.010	0.885 ± 0.013	0.878 ± 0.013	0.882 ± 0.010	0.764 ± 0.019
RF	1.000 ± 0.001	1.000 ± 0.000	1.000 ± 0.001	1.000 ± 0.001	1.000 ± 0.001	0.881 ± 0.005	0.893 ± 0.007	0.865 ± 0.008	0.879 ± 0.005	0.762 ± 0.010
Stack.	0.997 ± 0.003	0.999 ± 0.002	0.996 ± 0.004	0.997 ± 0.003	0.995 ± 0.006	0.888 ± 0.004	0.898 ± 0.008	0.876 ± 0.010	0.887 ± 0.005	0.777 ± 0.009

rfe

	Train Dataset					Test Dataset				
	Acc.	Prec.	Recall	F1	MCC	Acc.	Prec.	Recall	F1	MCC
SVC	0.962 ± 0.017	0.963 ± 0.022	0.962 ± 0.012	0.962 ± 0.017	0.925 ± 0.035	0.885 ± 0.009	0.890 ± 0.010	0.878 ± 0.013	0.884 ± 0.009	0.770 ± 0.018
LR	0.947 ± 0.031	0.948 ± 0.028	0.946 ± 0.034	0.947 ± 0.031	0.894 ± 0.061	0.879 ± 0.007	0.896 ± 0.008	0.858 ± 0.015	0.877 ± 0.008	0.759 ± 0.013
MLP	0.980 ± 0.012	0.990 ± 0.008	0.970 ± 0.021	0.980 ± 0.012	0.961 ± 0.024	0.872 ± 0.008	0.888 ± 0.025	0.852 ± 0.031	0.869 ± 0.009	0.745 ± 0.016
kNN	1.000 ± 0.000	1.000 ± 0.000	1.000 ± 0.000	1.000 ± 0.000	1.000 ± 0.000	0.879 ± 0.004	0.884 ± 0.006	0.873 ± 0.011	0.878 ± 0.004	0.759 ± 0.008
RF	1.000 ± 0.001	1.000 ± 0.000	0.999 ± 0.002	1.000 ± 0.001	0.999 ± 0.002	0.883 ± 0.013	0.903 ± 0.021	0.859 ± 0.016	0.881 ± 0.012	0.768 ± 0.025
Stack.	0.996 ± 0.004	0.998 ± 0.002	0.994 ± 0.005	0.996 ± 0.004	0.992 ± 0.007	0.892 ± 0.007	0.901 ± 0.013	0.881 ± 0.010	0.891 ± 0.006	0.784 ± 0.014

no

	Train Dataset					Test Dataset				
	Acc.	Prec.	Recall	F1	MCC	Acc.	Prec.	Recall	F1	MCC
SVC	0.979 ± 0.017	0.982 ± 0.018	0.975 ± 0.016	0.979 ± 0.017	0.957 ± 0.033	0.884 ± 0.007	0.887 ± 0.008	0.880 ± 0.018	0.883 ± 0.008	0.768 ± 0.014
LR	0.988 ± 0.010	0.991 ± 0.009	0.984 ± 0.011	0.988 ± 0.010	0.975 ± 0.020	0.878 ± 0.004	0.889 ± 0.007	0.863 ± 0.000	0.876 ± 0.003	0.756 ± 0.008
MLP	0.994 ± 0.010	0.998 ± 0.003	0.990 ± 0.017	0.994 ± 0.010	0.989 ± 0.019	0.865 ± 0.003	0.889 ± 0.007	0.834 ± 0.014	0.860 ± 0.005	0.731 ± 0.006
kNN	1.000 ± 0.000	1.000 ± 0.000	1.000 ± 0.000	1.000 ± 0.000	1.000 ± 0.000	0.883 ± 0.002	0.897 ± 0.006	0.866 ± 0.003	0.881 ± 0.001	0.766 ± 0.003
RF	1.000 ± 0.000	1.000 ± 0.000	1.000 ± 0.000	1.000 ± 0.000	1.000 ± 0.000	0.885 ± 0.006	0.907 ± 0.003	0.858 ± 0.013	0.882 ± 0.007	0.771 ± 0.012
Stack.	0.998 ± 0.002	1.000 ± 0.001	0.996 ± 0.003	0.998 ± 0.002	0.996 ± 0.003	0.884 ± 0.009	0.893 ± 0.010	0.873 ± 0.009	0.883 ± 0.009	0.768 ± 0.018

Prottrans

kb

	Train Dataset					Test Dataset				
	Acc.	Prec.	Recall	F1	MCC	Acc.	Prec.	Recall	F1	MCC
SVC	0.978 ± 0.022	0.976 ± 0.025	0.980 ± 0.019	0.978 ± 0.022	0.956 ± 0.044	0.883 ± 0.014	0.882 ± 0.006	0.883 ± 0.025	0.883 ± 0.015	0.765 ± 0.028
LR	0.909 ± 0.009	0.899 ± 0.008	0.920 ± 0.009	0.910 ± 0.009	0.817 ± 0.017	0.880 ± 0.005	0.877 ± 0.005	0.885 ± 0.005	0.881 ± 0.005	0.760 ± 0.010
MLP	0.951 ± 0.036	0.944 ± 0.046	0.959 ± 0.024	0.951 ± 0.035	0.902 ± 0.071	0.886 ± 0.005	0.890 ± 0.011	0.882 ± 0.023	0.886 ± 0.007	0.773 ± 0.009
kNN	1.000 ± 0.000	1.000 ± 0.000	1.000 ± 0.000	1.000 ± 0.000	1.000 ± 0.000	0.873 ± 0.010	0.860 ± 0.012	0.891 ± 0.006	0.875 ± 0.009	0.746 ± 0.020
RF	1.000 ± 0.001	1.000 ± 0.001	0.999 ± 0.001	1.000 ± 0.001	0.999 ± 0.002	0.876 ± 0.009	0.883 ± 0.006	0.868 ± 0.017	0.875 ± 0.010	0.753 ± 0.019
Stack.	0.994 ± 0.006	0.997 ± 0.004	0.992 ± 0.008	0.994 ± 0.006	0.989 ± 0.012	0.886 ± 0.003	0.886 ± 0.006	0.886 ± 0.005	0.886 ± 0.003	0.772 ± 0.007

pca

	Train Dataset					Test Dataset				
	Acc.	Prec.	Recall	F1	MCC	Acc.	Prec.	Recall	F1	MCC
SVC	0.975 ± 0.008	0.972 ± 0.009	0.978 ± 0.008	0.975 ± 0.008	0.950 ± 0.017	0.882 ± 0.006	0.889 ± 0.007	0.872 ± 0.006	0.880 ± 0.006	0.763 ± 0.013
LR	0.935 ± 0.022	0.925 ± 0.024	0.947 ± 0.018	0.936 ± 0.021	0.870 ± 0.043	0.875 ± 0.005	0.883 ± 0.003	0.865 ± 0.013	0.874 ± 0.006	0.751 ± 0.009
MLP	0.994 ± 0.010	0.995 ± 0.008	0.994 ± 0.012	0.994 ± 0.010	0.989 ± 0.020	0.874 ± 0.010	0.880 ± 0.009	0.865 ± 0.015	0.872 ± 0.011	0.747 ± 0.021
kNN	1.000 ± 0.000	1.000 ± 0.000	1.000 ± 0.000	1.000 ± 0.000	1.000 ± 0.000	0.872 ± 0.014	0.862 ± 0.015	0.885 ± 0.013	0.873 ± 0.014	0.744 ± 0.029
RF	1.000 ± 0.001	1.000 ± 0.001	0.999 ± 0.002	1.000 ± 0.001	0.999 ± 0.002	0.852 ± 0.008	0.851 ± 0.023	0.854 ± 0.022	0.852 ± 0.007	0.704 ± 0.017
Stack.	0.989 ± 0.005	0.990 ± 0.007	0.988 ± 0.004	0.989 ± 0.005	0.979 ± 0.011	0.889 ± 0.007	0.895 ± 0.007	0.881 ± 0.010	0.888 ± 0.008	0.778 ± 0.015

sfm

	Train Dataset					Test Dataset				
	Acc.	Prec.	Recall	F1	MCC	Acc.	Prec.	Recall	F1	MCC
SVC	0.945 ± 0.028	0.935 ± 0.035	0.956 ± 0.020	0.946 ± 0.027	0.890 ± 0.055	0.881 ± 0.010	0.876 ± 0.008	0.888 ± 0.016	0.882 ± 0.011	0.762 ± 0.021
LR	0.902 ± 0.011	0.891 ± 0.013	0.915 ± 0.009	0.903 ± 0.010	0.803 ± 0.021	0.873 ± 0.011	0.873 ± 0.013	0.873 ± 0.014	0.873 ± 0.011	0.745 ± 0.022
MLP	0.941 ± 0.046	0.934 ± 0.051	0.950 ± 0.039	0.942 ± 0.045	0.882 ± 0.091	0.873 ± 0.017	0.875 ± 0.012	0.869 ± 0.031	0.872 ± 0.019	0.746 ± 0.033
kNN	1.000 ± 0.000	1.000 ± 0.000	1.000 ± 0.000	1.000 ± 0.000	1.000 ± 0.000	0.876 ± 0.005	0.865 ± 0.012	0.892 ± 0.007	0.878 ± 0.004	0.752 ± 0.010
RF	0.999 ± 0.002	0.999 ± 0.002	0.999 ± 0.002	0.999 ± 0.002	0.997 ± 0.004	0.876 ± 0.007	0.881 ± 0.007	0.870 ± 0.013	0.876 ± 0.007	0.753 ± 0.013
Stack.	0.990 ± 0.006	0.993 ± 0.005	0.986 ± 0.007	0.990 ± 0.006	0.979 ± 0.012	0.891 ± 0.006	0.888 ± 0.005	0.893 ± 0.009	0.891 ± 0.006	0.781 ± 0.012

rfe

	Train Dataset					Test Dataset				
	Acc.	Prec.	Recall	F1	MCC	Acc.	Prec.	Recall	F1	MCC
SVC	0.957 ± 0.011	0.952 ± 0.011	0.963 ± 0.011	0.957 ± 0.010	0.914 ± 0.021	0.890 ± 0.007	0.891 ± 0.015	0.889 ± 0.011	0.890 ± 0.006	0.780 ± 0.014
LR	0.897 ± 0.013	0.886 ± 0.012	0.912 ± 0.014	0.899 ± 0.013	0.795 ± 0.026	0.874 ± 0.012	0.875 ± 0.012	0.872 ± 0.016	0.873 ± 0.013	0.747 ± 0.025
MLP	0.934 ± 0.044	0.924 ± 0.047	0.946 ± 0.040	0.935 ± 0.044	0.868 ± 0.089	0.877 ± 0.016	0.875 ± 0.023	0.880 ± 0.010	0.878 ± 0.014	0.755 ± 0.031
kNN	1.000 ± 0.000	1.000 ± 0.000	1.000 ± 0.000	1.000 ± 0.000	1.000 ± 0.000	0.878 ± 0.019	0.861 ± 0.022	0.902 ± 0.015	0.881 ± 0.018	0.757 ± 0.037
RF	0.999 ± 0.001	1.000 ± 0.001	0.999 ± 0.002	0.999 ± 0.001	0.998 ± 0.002	0.881 ± 0.010	0.883 ± 0.009	0.878 ± 0.014	0.881 ± 0.010	0.762 ± 0.020
Stack.	0.987 ± 0.006	0.990 ± 0.006	0.985 ± 0.006	0.987 ± 0.006	0.975 ± 0.012	0.893 ± 0.003	0.895 ± 0.009	0.891 ± 0.006	0.893 ± 0.003	0.786 ± 0.007

no

	Train Dataset					Test Dataset				
	Acc.	Prec.	Recall	F1	MCC	Acc.	Prec.	Recall	F1	MCC
SVC	0.972 ± 0.022	0.969 ± 0.023	0.975 ± 0.020	0.972 ± 0.022	0.944 ± 0.043	0.879 ± 0.007	0.884 ± 0.009	0.872 ± 0.006	0.878 ± 0.007	0.758 ± 0.015
LR	0.933 ± 0.010	0.926 ± 0.012	0.942 ± 0.009	0.934 ± 0.010	0.867 ± 0.020	0.875 ± 0.000	0.883 ± 0.004	0.864 ± 0.005	0.874 ± 0.001	0.750 ± 0.000
MLP	0.977 ± 0.024	0.971 ± 0.035	0.984 ± 0.014	0.977 ± 0.024	0.954 ± 0.048	0.872 ± 0.005	0.873 ± 0.016	0.870 ± 0.020	0.871 ± 0.006	0.744 ± 0.010
kNN	1.000 ± 0.000	1.000 ± 0.000	1.000 ± 0.000	1.000 ± 0.000	1.000 ± 0.000	0.870 ± 0.021	0.857 ± 0.020	0.888 ± 0.022	0.872 ± 0.021	0.740 ± 0.043
RF	0.999 ± 0.001	1.000 ± 0.001	0.999 ± 0.002	0.999 ± 0.001	0.998 ± 0.002	0.875 ± 0.004	0.884 ± 0.006	0.864 ± 0.009	0.874 ± 0.005	0.751 ± 0.009
Stack.	0.991 ± 0.007	0.992 ± 0.007	0.990 ± 0.008	0.991 ± 0.007	0.982 ± 0.014	0.895 ± 0.009	0.900 ± 0.012	0.890 ± 0.007	0.895 ± 0.009	0.791 ± 0.019

plusRNN

kb

	Train Dataset					Test Dataset				
	Acc.	Prec.	Recall	F1	MCC	Acc.	Prec.	Recall	F1	MCC
SVC	0.958 ± 0.023	0.950 ± 0.031	0.966 ± 0.013	0.958 ± 0.022	0.916 ± 0.045	0.874 ± 0.008	0.870 ± 0.012	0.880 ± 0.005	0.875 ± 0.007	0.748 ± 0.015
LR	0.898 ± 0.004	0.888 ± 0.003	0.912 ± 0.005	0.900 ± 0.004	0.797 ± 0.008	0.870 ± 0.002	0.875 ± 0.002	0.864 ± 0.005	0.869 ± 0.002	0.741 ± 0.004
MLP	0.926 ± 0.032	0.917 ± 0.041	0.938 ± 0.026	0.927 ± 0.031	0.853 ± 0.064	0.862 ± 0.005	0.862 ± 0.013	0.862 ± 0.019	0.862 ± 0.006	0.724 ± 0.010
kNN	1.000 ± 0.000	1.000 ± 0.000	1.000 ± 0.000	1.000 ± 0.000	1.000 ± 0.000	0.863 ± 0.009	0.827 ± 0.014	0.918 ± 0.014	0.870 ± 0.008	0.730 ± 0.017
RF	1.000 ± 0.001	0.999 ± 0.002	1.000 ± 0.000	1.000 ± 0.001	0.999 ± 0.002	0.875 ± 0.012	0.860 ± 0.012	0.894 ± 0.015	0.877 ± 0.012	0.750 ± 0.025
Stack.	0.993 ± 0.003	0.995 ± 0.003	0.990 ± 0.003	0.993 ± 0.003	0.985 ± 0.006	0.888 ± 0.004	0.885 ± 0.003	0.892 ± 0.007	0.888 ± 0.005	0.776 ± 0.009

pca

	Train Dataset					Test Dataset				
	Acc.	Prec.	Recall	F1	MCC	Acc.	Prec.	Recall	F1	MCC
SVC	0.992 ± 0.010	0.991 ± 0.012	0.993 ± 0.008	0.992 ± 0.010	0.983 ± 0.020	0.881 ± 0.004	0.878 ± 0.003	0.886 ± 0.008	0.882 ± 0.005	0.762 ± 0.009
LR	0.936 ± 0.016	0.929 ± 0.021	0.944 ± 0.011	0.936 ± 0.016	0.872 ± 0.032	0.879 ± 0.006	0.883 ± 0.010	0.874 ± 0.006	0.878 ± 0.006	0.758 ± 0.013
MLP	0.972 ± 0.028	0.968 ± 0.032	0.977 ± 0.024	0.972 ± 0.028	0.944 ± 0.057	0.881 ± 0.006	0.886 ± 0.010	0.875 ± 0.005	0.880 ± 0.005	0.762 ± 0.011
kNN	1.000 ± 0.000	1.000 ± 0.000	1.000 ± 0.000	1.000 ± 0.000	1.000 ± 0.000	0.862 ± 0.010	0.843 ± 0.012	0.889 ± 0.008	0.865 ± 0.010	0.725 ± 0.021
RF	0.990 ± 0.020	0.986 ± 0.028	0.996 ± 0.011	0.991 ± 0.020	0.981 ± 0.040	0.812 ± 0.025	0.828 ± 0.032	0.790 ± 0.049	0.808 ± 0.028	0.626 ± 0.049
Stack.	0.994 ± 0.007	0.995 ± 0.007	0.993 ± 0.007	0.994 ± 0.007	0.988 ± 0.014	0.888 ± 0.005	0.895 ± 0.007	0.878 ± 0.005	0.887 ± 0.005	0.776 ± 0.010

sfm

	Train Dataset					Test Dataset				
	Acc.	Prec.	Recall	F1	MCC	Acc.	Prec.	Recall	F1	MCC
SVC	0.966 ± 0.016	0.960 ± 0.017	0.973 ± 0.016	0.966 ± 0.016	0.932 ± 0.033	0.873 ± 0.005	0.872 ± 0.014	0.875 ± 0.009	0.873 ± 0.004	0.746 ± 0.009
LR	0.899 ± 0.018	0.887 ± 0.019	0.913 ± 0.017	0.900 ± 0.018	0.798 ± 0.036	0.865 ± 0.013	0.871 ± 0.016	0.856 ± 0.012	0.863 ± 0.013	0.729 ± 0.026
MLP	0.941 ± 0.023	0.936 ± 0.027	0.948 ± 0.018	0.941 ± 0.022	0.882 ± 0.046	0.872 ± 0.006	0.882 ± 0.017	0.859 ± 0.016	0.871 ± 0.005	0.745 ± 0.011
kNN	1.000 ± 0.000	1.000 ± 0.000	1.000 ± 0.000	1.000 ± 0.000	1.000 ± 0.000	0.857 ± 0.014	0.824 ± 0.018	0.908 ± 0.012	0.864 ± 0.012	0.718 ± 0.027
RF	0.999 ± 0.001	0.998 ± 0.002	1.000 ± 0.000	0.999 ± 0.001	0.998 ± 0.002	0.872 ± 0.007	0.861 ± 0.009	0.888 ± 0.010	0.874 ± 0.007	0.745 ± 0.013
Stack.	0.995 ± 0.003	0.997 ± 0.002	0.993 ± 0.005	0.995 ± 0.003	0.990 ± 0.006	0.886 ± 0.003	0.882 ± 0.010	0.892 ± 0.010	0.886 ± 0.003	0.772 ± 0.007

rfe

	Train Dataset					Test Dataset				
	Acc.	Prec.	Recall	F1	MCC	Acc.	Prec.	Recall	F1	MCC
SVC	0.962 ± 0.024	0.956 ± 0.029	0.969 ± 0.018	0.962 ± 0.024	0.924 ± 0.048	0.882 ± 0.006	0.881 ± 0.006	0.882 ± 0.011	0.882 ± 0.006	0.763 ± 0.011
LR	0.910 ± 0.024	0.902 ± 0.027	0.919 ± 0.020	0.911 ± 0.024	0.820 ± 0.048	0.868 ± 0.011	0.871 ± 0.010	0.863 ± 0.013	0.867 ± 0.011	0.736 ± 0.022
MLP	0.948 ± 0.024	0.940 ± 0.024	0.957 ± 0.029	0.948 ± 0.023	0.895 ± 0.047	0.878 ± 0.012	0.882 ± 0.010	0.874 ± 0.026	0.878 ± 0.013	0.757 ± 0.023
kNN	1.000 ± 0.000	1.000 ± 0.000	1.000 ± 0.000	1.000 ± 0.000	1.000 ± 0.000	0.861 ± 0.008	0.833 ± 0.005	0.904 ± 0.017	0.867 ± 0.008	0.725 ± 0.016
RF	0.998 ± 0.003	0.997 ± 0.005	0.999 ± 0.001	0.998 ± 0.003	0.996 ± 0.007	0.875 ± 0.009	0.860 ± 0.011	0.896 ± 0.013	0.878 ± 0.009	0.751 ± 0.018
Stack.	0.992 ± 0.005	0.992 ± 0.005	0.992 ± 0.006	0.992 ± 0.005	0.984 ± 0.011	0.885 ± 0.018	0.884 ± 0.025	0.887 ± 0.009	0.885 ± 0.017	0.770 ± 0.035

no

	Train Dataset					Test Dataset				
	Acc.	Prec.	Recall	F1	MCC	Acc.	Prec.	Recall	F1	MCC
SVC	0.977 ± 0.022	0.975 ± 0.027	0.981 ± 0.017	0.978 ± 0.022	0.955 ± 0.043	0.882 ± 0.013	0.884 ± 0.020	0.878 ± 0.010	0.881 ± 0.012	0.763 ± 0.026
LR	0.935 ± 0.028	0.928 ± 0.036	0.944 ± 0.018	0.936 ± 0.027	0.871 ± 0.056	0.874 ± 0.003	0.878 ± 0.012	0.869 ± 0.010	0.873 ± 0.003	0.748 ± 0.007
MLP	0.960 ± 0.026	0.956 ± 0.017	0.964 ± 0.036	0.960 ± 0.026	0.920 ± 0.051	0.879 ± 0.006	0.881 ± 0.013	0.876 ± 0.013	0.878 ± 0.006	0.758 ± 0.012
kNN	0.973 ± 0.074	0.967 ± 0.093	0.983 ± 0.046	0.975 ± 0.070	0.948 ± 0.146	0.859 ± 0.009	0.829 ± 0.022	0.905 ± 0.016	0.865 ± 0.006	0.721 ± 0.015
RF	1.000 ± 0.001	1.000 ± 0.001	1.000 ± 0.000	1.000 ± 0.001	1.000 ± 0.001	0.869 ± 0.013	0.856 ± 0.021	0.889 ± 0.008	0.872 ± 0.012	0.739 ± 0.026
Stack.	0.994 ± 0.004	0.996 ± 0.003	0.993 ± 0.005	0.994 ± 0.004	0.989 ± 0.008	0.888 ± 0.006	0.895 ± 0.010	0.880 ± 0.009	0.887 ± 0.006	0.777 ± 0.013

ProtT5

kb

	Train Dataset					Test Dataset				
	Acc.	Prec.	Recall	F1	MCC	Acc.	Prec.	Recall	F1	MCC
SVC	0.809 ± 0.022	0.792 ± 0.020	0.842 ± 0.023	0.816 ± 0.021	0.618 ± 0.044	0.674 ± 0.005	0.662 ± 0.010	0.719 ± 0.015	0.689 ± 0.003	0.348 ± 0.010
LR	0.761 ± 0.015	0.752 ± 0.016	0.786 ± 0.012	0.768 ± 0.014	0.522 ± 0.030	0.661 ± 0.002	0.662 ± 0.005	0.668 ± 0.011	0.665 ± 0.004	0.323 ± 0.003
MLP	0.762 ± 0.020	0.745 ± 0.019	0.802 ± 0.032	0.773 ± 0.020	0.525 ± 0.040	0.666 ± 0.009	0.658 ± 0.003	0.702 ± 0.032	0.679 ± 0.015	0.333 ± 0.018
kNN	0.897 ± 0.174	0.891 ± 0.185	0.913 ± 0.145	0.901 ± 0.166	0.794 ± 0.347	0.680 ± 0.011	0.674 ± 0.010	0.708 ± 0.010	0.691 ± 0.010	0.361 ± 0.021
RF	0.991 ± 0.011	0.984 ± 0.019	0.998 ± 0.002	0.991 ± 0.011	0.982 ± 0.021	0.684 ± 0.011	0.684 ± 0.015	0.694 ± 0.016	0.689 ± 0.010	0.368 ± 0.023
Stack.	0.877 ± 0.051	0.869 ± 0.054	0.890 ± 0.043	0.879 ± 0.049	0.754 ± 0.101	0.684 ± 0.006	0.680 ± 0.009	0.703 ± 0.004	0.691 ± 0.004	0.367 ± 0.013

pca

	Train Dataset					Test Dataset				
	Acc.	Prec.	Recall	F1	MCC	Acc.	Prec.	Recall	F1	MCC
SVC	0.821 ± 0.063	0.804 ± 0.061	0.852 ± 0.059	0.827 ± 0.060	0.642 ± 0.125	0.688 ± 0.006	0.676 ± 0.004	0.729 ± 0.013	0.702 ± 0.008	0.377 ± 0.013
LR	0.735 ± 0.011	0.727 ± 0.010	0.760 ± 0.011	0.743 ± 0.010	0.470 ± 0.021	0.657 ± 0.008	0.649 ± 0.007	0.698 ± 0.008	0.672 ± 0.008	0.315 ± 0.016
MLP	0.816 ± 0.122	0.805 ± 0.127	0.840 ± 0.109	0.822 ± 0.118	0.632 ± 0.244	0.667 ± 0.019	0.667 ± 0.022	0.676 ± 0.013	0.672 ± 0.017	0.334 ± 0.039
kNN	0.950 ± 0.136	0.944 ± 0.152	0.963 ± 0.098	0.953 ± 0.127	0.900 ± 0.271	0.677 ± 0.007	0.672 ± 0.004	0.700 ± 0.025	0.686 ± 0.012	0.354 ± 0.014
RF	0.892 ± 0.045	0.857 ± 0.048	0.943 ± 0.033	0.898 ± 0.041	0.787 ± 0.089	0.693 ± 0.017	0.675 ± 0.016	0.753 ± 0.024	0.712 ± 0.017	0.387 ± 0.035
Stack.	0.883 ± 0.069	0.876 ± 0.070	0.896 ± 0.063	0.886 ± 0.067	0.767 ± 0.138	0.678 ± 0.004	0.675 ± 0.007	0.696 ± 0.009	0.686 ± 0.004	0.357 ± 0.009

sfm

	Train Dataset					Test Dataset				
	Acc.	Prec.	Recall	F1	MCC	Acc.	Prec.	Recall	F1	MCC
SVC	0.795 ± 0.064	0.777 ± 0.064	0.832 ± 0.054	0.804 ± 0.060	0.590 ± 0.127	0.676 ± 0.006	0.662 ± 0.007	0.730 ± 0.032	0.694 ± 0.013	0.355 ± 0.014
LR	0.754 ± 0.015	0.747 ± 0.017	0.775 ± 0.010	0.760 ± 0.013	0.508 ± 0.030	0.662 ± 0.013	0.659 ± 0.013	0.681 ± 0.020	0.670 ± 0.014	0.324 ± 0.026
MLP	0.737 ± 0.010	0.728 ± 0.010	0.765 ± 0.033	0.746 ± 0.015	0.475 ± 0.022	0.669 ± 0.012	0.662 ± 0.016	0.701 ± 0.024	0.681 ± 0.012	0.339 ± 0.024
kNN	0.807 ± 0.134	0.793 ± 0.144	0.842 ± 0.109	0.816 ± 0.128	0.615 ± 0.267	0.695 ± 0.016	0.689 ± 0.016	0.718 ± 0.016	0.703 ± 0.015	0.390 ± 0.031
RF	0.994 ± 0.007	0.990 ± 0.013	0.999 ± 0.001	0.994 ± 0.007	0.989 ± 0.015	0.694 ± 0.014	0.691 ± 0.011	0.708 ± 0.026	0.699 ± 0.017	0.387 ± 0.029
Stack.	0.843 ± 0.081	0.837 ± 0.084	0.856 ± 0.074	0.847 ± 0.079	0.686 ± 0.162	0.693 ± 0.012	0.688 ± 0.013	0.716 ± 0.008	0.702 ± 0.010	0.386 ± 0.023

rfe

	Train Dataset					Test Dataset				
	Acc.	Prec.	Recall	F1	MCC	Acc.	Prec.	Recall	F1	MCC
SVC	0.806 ± 0.047	0.789 ± 0.045	0.841 ± 0.043	0.814 ± 0.044	0.613 ± 0.094	0.673 ± 0.010	0.660 ± 0.006	0.722 ± 0.026	0.689 ± 0.014	0.347 ± 0.022
LR	0.746 ± 0.030	0.737 ± 0.029	0.772 ± 0.029	0.754 ± 0.029	0.492 ± 0.061	0.657 ± 0.018	0.654 ± 0.018	0.678 ± 0.029	0.666 ± 0.020	0.314 ± 0.037
MLP	0.760 ± 0.057	0.750 ± 0.059	0.788 ± 0.052	0.769 ± 0.054	0.522 ± 0.114	0.661 ± 0.012	0.657 ± 0.006	0.686 ± 0.038	0.671 ± 0.019	0.323 ± 0.024
kNN	0.901 ± 0.166	0.895 ± 0.177	0.916 ± 0.140	0.905 ± 0.159	0.803 ± 0.332	0.682 ± 0.021	0.679 ± 0.020	0.701 ± 0.027	0.690 ± 0.022	0.364 ± 0.043
RF	0.969 ± 0.068	0.963 ± 0.072	0.978 ± 0.060	0.970 ± 0.066	0.939 ± 0.135	0.683 ± 0.010	0.680 ± 0.013	0.702 ± 0.010	0.691 ± 0.008	0.366 ± 0.021
Stack.	0.871 ± 0.107	0.865 ± 0.112	0.884 ± 0.095	0.874 ± 0.104	0.743 ± 0.213	0.685 ± 0.017	0.680 ± 0.014	0.706 ± 0.025	0.693 ± 0.018	0.369 ± 0.033

no

	Train Dataset					Test Dataset				
	Acc.	Prec.	Recall	F1	MCC	Acc.	Prec.	Recall	F1	MCC
SVC	0.796 ± 0.026	0.777 ± 0.023	0.836 ± 0.027	0.805 ± 0.025	0.594 ± 0.052	0.679 ± 0.008	0.668 ± 0.007	0.723 ± 0.013	0.694 ± 0.008	0.360 ± 0.015
LR	0.751 ± 0.024	0.742 ± 0.024	0.777 ± 0.020	0.759 ± 0.022	0.503 ± 0.047	0.661 ± 0.007	0.656 ± 0.011	0.689 ± 0.018	0.672 ± 0.007	0.323 ± 0.014
MLP	0.764 ± 0.011	0.760 ± 0.031	0.782 ± 0.074	0.769 ± 0.022	0.531 ± 0.024	0.664 ± 0.011	0.662 ± 0.021	0.685 ± 0.085	0.671 ± 0.032	0.331 ± 0.024
kNN	0.903 ± 0.164	0.899 ± 0.171	0.913 ± 0.145	0.906 ± 0.158	0.806 ± 0.327	0.695 ± 0.021	0.688 ± 0.019	0.721 ± 0.024	0.704 ± 0.021	0.390 ± 0.041
RF	0.988 ± 0.015	0.982 ± 0.021	0.994 ± 0.008	0.988 ± 0.015	0.976 ± 0.030	0.680 ± 0.013	0.680 ± 0.011	0.689 ± 0.028	0.684 ± 0.017	0.360 ± 0.027
Stack.	0.868 ± 0.067	0.858 ± 0.071	0.886 ± 0.059	0.872 ± 0.065	0.737 ± 0.134	0.688 ± 0.016	0.684 ± 0.017	0.708 ± 0.019	0.695 ± 0.016	0.376 ± 0.032

Bepler

kb

	Train Dataset					Test Dataset				
	Acc.	Prec.	Recall	F1	MCC	Acc.	Prec.	Recall	F1	MCC
SVC	0.989 ± 0.016	0.989 ± 0.018	0.988 ± 0.015	0.989 ± 0.016	0.977 ± 0.033	0.836 ± 0.004	0.845 ± 0.003	0.823 ± 0.011	0.834 ± 0.006	0.672 ± 0.009
LR	0.889 ± 0.004	0.892 ± 0.005	0.885 ± 0.005	0.889 ± 0.005	0.779 ± 0.009	0.867 ± 0.008	0.845 ± 0.009	0.900 ± 0.009	0.872 ± 0.008	0.736 ± 0.016
MLP	0.989 ± 0.023	0.990 ± 0.025	0.989 ± 0.022	0.989 ± 0.023	0.979 ± 0.046	0.857 ± 0.007	0.867 ± 0.009	0.842 ± 0.018	0.855 ± 0.008	0.714 ± 0.013
kNN	1.000 ± 0.000	1.000 ± 0.000	1.000 ± 0.000	1.000 ± 0.000	1.000 ± 0.000	0.763 ± 0.005	0.738 ± 0.005	0.814 ± 0.012	0.774 ± 0.006	0.528 ± 0.010
RF	1.000 ± 0.000	1.000 ± 0.000	1.000 ± 0.000	1.000 ± 0.000	1.000 ± 0.000	0.897 ± 0.009	0.873 ± 0.016	0.928 ± 0.006	0.900 ± 0.008	0.795 ± 0.018
Stack.	0.998 ± 0.002	0.998 ± 0.004	0.999 ± 0.001	0.998 ± 0.002	0.997 ± 0.004	0.896 ± 0.008	0.893 ± 0.012	0.900 ± 0.014	0.896 ± 0.008	0.792 ± 0.016

pca

	Train Dataset					Test Dataset				
	Acc.	Prec.	Recall	F1	MCC	Acc.	Prec.	Recall	F1	MCC
SVC	0.959 ± 0.049	0.953 ± 0.054	0.966 ± 0.042	0.959 ± 0.048	0.918 ± 0.097	0.818 ± 0.008	0.814 ± 0.013	0.825 ± 0.031	0.820 ± 0.011	0.637 ± 0.017
LR	0.889 ± 0.005	0.891 ± 0.004	0.887 ± 0.005	0.889 ± 0.005	0.779 ± 0.009	0.868 ± 0.008	0.847 ± 0.008	0.899 ± 0.007	0.872 ± 0.007	0.738 ± 0.015
MLP	1.000 ± 0.000	1.000 ± 0.000	1.000 ± 0.000	1.000 ± 0.000	1.000 ± 0.000	0.864 ± 0.016	0.869 ± 0.023	0.858 ± 0.015	0.863 ± 0.015	0.729 ± 0.032
kNN	1.000 ± 0.000	1.000 ± 0.000	1.000 ± 0.000	1.000 ± 0.000	1.000 ± 0.000	0.769 ± 0.015	0.738 ± 0.014	0.836 ± 0.015	0.784 ± 0.014	0.544 ± 0.031
RF	1.000 ± 0.001	0.999 ± 0.002	1.000 ± 0.000	1.000 ± 0.001	0.999 ± 0.002	0.839 ± 0.018	0.834 ± 0.020	0.846 ± 0.020	0.840 ± 0.017	0.678 ± 0.036
Stack.	0.999 ± 0.002	1.000 ± 0.001	0.999 ± 0.002	0.999 ± 0.002	0.999 ± 0.003	0.884 ± 0.007	0.881 ± 0.008	0.889 ± 0.013	0.885 ± 0.008	0.769 ± 0.014

sfm

	Train Dataset					Test Dataset				
	Acc.	Prec.	Recall	F1	MCC	Acc.	Prec.	Recall	F1	MCC
SVC	0.978 ± 0.013	0.977 ± 0.014	0.979 ± 0.013	0.978 ± 0.013	0.957 ± 0.026	0.833 ± 0.007	0.842 ± 0.003	0.821 ± 0.014	0.831 ± 0.008	0.667 ± 0.014
LR	0.890 ± 0.004	0.893 ± 0.004	0.887 ± 0.004	0.890 ± 0.004	0.780 ± 0.008	0.869 ± 0.006	0.847 ± 0.005	0.901 ± 0.009	0.873 ± 0.006	0.739 ± 0.012
MLP	0.958 ± 0.046	0.964 ± 0.041	0.952 ± 0.053	0.958 ± 0.047	0.917 ± 0.092	0.861 ± 0.009	0.859 ± 0.015	0.864 ± 0.023	0.861 ± 0.010	0.722 ± 0.018
kNN	1.000 ± 0.000	1.000 ± 0.000	1.000 ± 0.000	1.000 ± 0.000	1.000 ± 0.000	0.761 ± 0.005	0.737 ± 0.004	0.810 ± 0.015	0.772 ± 0.007	0.524 ± 0.011
RF	1.000 ± 0.000	1.000 ± 0.001	1.000 ± 0.000	1.000 ± 0.000	1.000 ± 0.001	0.895 ± 0.011	0.877 ± 0.014	0.919 ± 0.013	0.897 ± 0.011	0.791 ± 0.022
Stack.	0.994 ± 0.004	0.994 ± 0.004	0.993 ± 0.005	0.994 ± 0.004	0.987 ± 0.008	0.895 ± 0.005	0.890 ± 0.008	0.902 ± 0.010	0.896 ± 0.005	0.791 ± 0.010

rfe

	Train Dataset					Test Dataset				
	Acc.	Prec.	Recall	F1	MCC	Acc.	Prec.	Recall	F1	MCC
SVC	0.949 ± 0.031	0.939 ± 0.039	0.961 ± 0.021	0.950 ± 0.030	0.898 ± 0.061	0.845 ± 0.013	0.841 ± 0.010	0.852 ± 0.024	0.846 ± 0.015	0.691 ± 0.027
LR	0.866 ± 0.008	0.860 ± 0.008	0.874 ± 0.009	0.867 ± 0.008	0.733 ± 0.016	0.863 ± 0.005	0.850 ± 0.006	0.881 ± 0.011	0.865 ± 0.006	0.726 ± 0.011
MLP	0.899 ± 0.021	0.887 ± 0.022	0.914 ± 0.032	0.900 ± 0.022	0.799 ± 0.043	0.868 ± 0.013	0.851 ± 0.021	0.893 ± 0.010	0.872 ± 0.011	0.738 ± 0.025
kNN	1.000 ± 0.000	1.000 ± 0.000	1.000 ± 0.000	1.000 ± 0.000	1.000 ± 0.000	0.763 ± 0.007	0.734 ± 0.007	0.825 ± 0.010	0.777 ± 0.007	0.530 ± 0.015
RF	0.998 ± 0.002	0.997 ± 0.004	0.999 ± 0.001	0.998 ± 0.002	0.996 ± 0.005	0.895 ± 0.005	0.874 ± 0.008	0.924 ± 0.003	0.898 ± 0.005	0.792 ± 0.010
Stack.	0.984 ± 0.013	0.982 ± 0.017	0.986 ± 0.008	0.984 ± 0.013	0.967 ± 0.025	0.890 ± 0.008	0.880 ± 0.009	0.903 ± 0.009	0.891 ± 0.008	0.780 ± 0.016

no

	Train Dataset					Test Dataset				
	Acc.	Prec.	Recall	F1	MCC	Acc.	Prec.	Recall	F1	MCC
SVC	0.981 ± 0.009	0.979 ± 0.009	0.982 ± 0.009	0.981 ± 0.009	0.961 ± 0.017	0.835 ± 0.005	0.841 ± 0.004	0.826 ± 0.012	0.833 ± 0.007	0.670 ± 0.011
LR	0.892 ± 0.000	0.894 ± 0.000	0.889 ± 0.000	0.892 ± 0.000	0.784 ± 0.000	0.870 ± 0.000	0.849 ± 0.000	0.901 ± 0.000	0.874 ± 0.000	0.742 ± 0.000
MLP	0.951 ± 0.054	0.953 ± 0.062	0.950 ± 0.048	0.951 ± 0.053	0.903 ± 0.108	0.871 ± 0.004	0.876 ± 0.024	0.865 ± 0.032	0.870 ± 0.006	0.742 ± 0.008
kNN	1.000 ± 0.000	1.000 ± 0.000	1.000 ± 0.000	1.000 ± 0.000	1.000 ± 0.000	0.766 ± 0.003	0.741 ± 0.005	0.817 ± 0.003	0.777 ± 0.001	0.534 ± 0.005
RF	1.000 ± 0.000	1.000 ± 0.001	1.000 ± 0.000	1.000 ± 0.000	1.000 ± 0.001	0.888 ± 0.012	0.872 ± 0.020	0.910 ± 0.006	0.891 ± 0.010	0.777 ± 0.023
Stack.	0.996 ± 0.004	0.996 ± 0.005	0.997 ± 0.004	0.996 ± 0.004	0.993 ± 0.009	0.899 ± 0.005	0.892 ± 0.007	0.908 ± 0.011	0.899 ± 0.005	0.797 ± 0.010

TABLE S4

Models metrics averaging over all feature-selection methods

ESM1b

	Train Dataset					Test Dataset				
	Acc.	Prec.	Recall	F1	MCC	Acc.	Prec.	Recall	F1	MCC
SVC	0.976 ± 0.009	0.979 ± 0.009	0.973 ± 0.008	0.976 ± 0.009	0.952 ± 0.017	0.884 ± 0.005	0.887 ± 0.006	0.881 ± 0.007	0.884 ± 0.005	0.768 ± 0.009
LR	0.950 ± 0.015	0.951 ± 0.015	0.949 ± 0.015	0.950 ± 0.015	0.900 ± 0.029	0.876 ± 0.004	0.886 ± 0.007	0.863 ± 0.005	0.874 ± 0.004	0.752 ± 0.009
MLP	0.984 ± 0.010	0.986 ± 0.011	0.983 ± 0.009	0.984 ± 0.009	0.969 ± 0.019	0.871 ± 0.004	0.879 ± 0.009	0.861 ± 0.012	0.869 ± 0.005	0.742 ± 0.009
kNN	1.000 ± 0.000	1.000 ± 0.000	1.000 ± 0.000	1.000 ± 0.000	1.000 ± 0.000	0.881 ± 0.002	0.889 ± 0.005	0.871 ± 0.005	0.880 ± 0.002	0.762 ± 0.005
RF	1.000 ± 0.000	1.000 ± 0.000	1.000 ± 0.000	1.000 ± 0.000	1.000 ± 0.000	0.884 ± 0.004	0.900 ± 0.006	0.865 ± 0.007	0.882 ± 0.004	0.769 ± 0.008
Stack.	0.995 ± 0.002	0.997 ± 0.002	0.993 ± 0.002	0.995 ± 0.002	0.991 ± 0.003	0.888 ± 0.003	0.895 ± 0.004	0.880 ± 0.005	0.887 ± 0.003	0.777 ± 0.006

plusRNN

	Train Dataset					Test Dataset				
	Acc.	Prec.	Recall	F1	MCC	Acc.	Prec.	Recall	F1	MCC
SVC	0.971 ± 0.011	0.966 ± 0.013	0.976 ± 0.008	0.971 ± 0.010	0.942 ± 0.021	0.878 ± 0.004	0.877 ± 0.006	0.880 ± 0.004	0.879 ± 0.004	0.757 ± 0.008
LR	0.916 ± 0.013	0.907 ± 0.014	0.926 ± 0.010	0.917 ± 0.012	0.831 ± 0.025	0.871 ± 0.004	0.876 ± 0.005	0.865 ± 0.005	0.870 ± 0.004	0.742 ± 0.009
MLP	0.949 ± 0.014	0.943 ± 0.015	0.957 ± 0.013	0.950 ± 0.014	0.899 ± 0.028	0.874 ± 0.005	0.878 ± 0.007	0.869 ± 0.008	0.874 ± 0.005	0.749 ± 0.010
kNN	0.995 ± 0.015	0.993 ± 0.018	0.997 ± 0.009	0.995 ± 0.014	0.990 ± 0.029	0.860 ± 0.004	0.831 ± 0.007	0.905 ± 0.008	0.866 ± 0.004	0.724 ± 0.008
RF	0.997 ± 0.004	0.996 ± 0.006	0.999 ± 0.002	0.997 ± 0.004	0.995 ± 0.008	0.861 ± 0.015	0.853 ± 0.011	0.871 ± 0.025	0.862 ± 0.017	0.722 ± 0.030
Stack.	0.994 ± 0.002	0.995 ± 0.002	0.992 ± 0.002	0.994 ± 0.002	0.987 ± 0.004	0.887 ± 0.004	0.888 ± 0.006	0.886 ± 0.005	0.887 ± 0.004	0.774 ± 0.007

ProtT5

	Train Dataset					Test Dataset				
	Acc.	Prec.	Recall	F1	MCC	Acc.	Prec.	Recall	F1	MCC
SVC	0.805 ± 0.020	0.788 ± 0.020	0.840 ± 0.018	0.813 ± 0.019	0.612 ± 0.040	0.678 ± 0.004	0.666 ± 0.004	0.725 ± 0.009	0.694 ± 0.005	0.357 ± 0.009
LR	0.749 ± 0.010	0.741 ± 0.010	0.774 ± 0.009	0.757 ± 0.009	0.499 ± 0.019	0.660 ± 0.005	0.656 ± 0.005	0.683 ± 0.010	0.669 ± 0.005	0.320 ± 0.009
MLP	0.768 ± 0.029	0.758 ± 0.030	0.796 ± 0.031	0.776 ± 0.028	0.537 ± 0.058	0.666 ± 0.005	0.661 ± 0.007	0.690 ± 0.019	0.675 ± 0.009	0.332 ± 0.011
kNN	0.891 ± 0.069	0.884 ± 0.073	0.909 ± 0.057	0.896 ± 0.065	0.784 ± 0.137	0.686 ± 0.008	0.680 ± 0.007	0.710 ± 0.010	0.695 ± 0.008	0.372 ± 0.016
RF	0.967 ± 0.027	0.955 ± 0.033	0.983 ± 0.017	0.968 ± 0.025	0.935 ± 0.052	0.687 ± 0.006	0.682 ± 0.006	0.709 ± 0.016	0.695 ± 0.008	0.374 ± 0.013
Stack.	0.869 ± 0.032	0.861 ± 0.034	0.882 ± 0.029	0.872 ± 0.031	0.737 ± 0.065	0.686 ± 0.006	0.681 ± 0.006	0.706 ± 0.007	0.693 ± 0.006	0.371 ± 0.011

Prottrans

	Train Dataset					Test Dataset				
	Acc.	Prec.	Recall	F1	MCC	Acc.	Prec.	Recall	F1	MCC
SVC	0.965 ± 0.011	0.961 ± 0.013	0.970 ± 0.009	0.966 ± 0.010	0.931 ± 0.021	0.883 ± 0.004	0.885 ± 0.005	0.881 ± 0.007	0.883 ± 0.005	0.766 ± 0.009
LR	0.915 ± 0.011	0.906 ± 0.011	0.927 ± 0.010	0.916 ± 0.010	0.831 ± 0.021	0.875 ± 0.004	0.878 ± 0.004	0.872 ± 0.006	0.875 ± 0.004	0.751 ± 0.007
MLP	0.959 ± 0.019	0.953 ± 0.022	0.966 ± 0.016	0.960 ± 0.019	0.919 ± 0.038	0.876 ± 0.006	0.879 ± 0.007	0.873 ± 0.009	0.876 ± 0.006	0.753 ± 0.011
kNN	1.000 ± 0.000	1.000 ± 0.000	1.000 ± 0.000	1.000 ± 0.000	1.000 ± 0.000	0.874 ± 0.006	0.861 ± 0.007	0.891 ± 0.007	0.876 ± 0.006	0.748 ± 0.013
RF	0.999 ± 0.001	1.000 ± 0.001	0.999 ± 0.001	0.999 ± 0.001	0.998 ± 0.001	0.872 ± 0.007	0.877 ± 0.009	0.867 ± 0.008	0.872 ± 0.007	0.745 ± 0.013
Stack.	0.990 ± 0.003	0.992 ± 0.003	0.988 ± 0.003	0.990 ± 0.003	0.981 ± 0.006	0.891 ± 0.003	0.893 ± 0.004	0.888 ± 0.004	0.890 ± 0.003	0.782 ± 0.006

SeqVec

	Train Dataset					Test Dataset				
	Acc.	Prec.	Recall	F1	MCC	Acc.	Prec.	Recall	F1	MCC
SVC	0.997 ± 0.004	0.997 ± 0.003	0.997 ± 0.004	0.997 ± 0.004	0.995 ± 0.007	0.912 ± 0.004	0.920 ± 0.006	0.903 ± 0.005	0.912 ± 0.004	0.825 ± 0.009
LR	0.958 ± 0.013	0.956 ± 0.013	0.959 ± 0.013	0.958 ± 0.013	0.915 ± 0.026	0.886 ± 0.005	0.895 ± 0.006	0.875 ± 0.006	0.885 ± 0.005	0.773 ± 0.009
MLP	0.997 ± 0.004	0.997 ± 0.004	0.996 ± 0.005	0.997 ± 0.004	0.993 ± 0.008	0.891 ± 0.004	0.897 ± 0.007	0.883 ± 0.006	0.890 ± 0.004	0.782 ± 0.008
kNN	1.000 ± 0.000	1.000 ± 0.000	1.000 ± 0.000	1.000 ± 0.000	1.000 ± 0.000	0.898 ± 0.003	0.875 ± 0.006	0.928 ± 0.005	0.901 ± 0.003	0.797 ± 0.007
RF	1.000 ± 0.001	1.000 ± 0.001	1.000 ± 0.001	1.000 ± 0.001	0.999 ± 0.001	0.894 ± 0.007	0.905 ± 0.008	0.880 ± 0.007	0.892 ± 0.006	0.788 ± 0.013
Stack.	1.000 ± 0.001	1.000 ± 0.000	1.000 ± 0.001	1.000 ± 0.001	0.999 ± 0.001	0.918 ± 0.005	0.928 ± 0.007	0.906 ± 0.005	0.917 ± 0.004	0.836 ± 0.009

Bepler

	Train Dataset					Test Dataset				
	Acc.	Prec.	Recall	F1	MCC	Acc.	Prec.	Recall	F1	MCC
SVC	0.971 ± 0.014	0.967 ± 0.017	0.975 ± 0.011	0.971 ± 0.014	0.942 ± 0.028	0.834 ± 0.006	0.837 ± 0.007	0.829 ± 0.010	0.833 ± 0.006	0.667 ± 0.012
LR	0.885 ± 0.006	0.886 ± 0.008	0.884 ± 0.004	0.885 ± 0.005	0.771 ± 0.012	0.868 ± 0.003	0.848 ± 0.003	0.896 ± 0.005	0.871 ± 0.003	0.736 ± 0.006
MLP	0.960 ± 0.024	0.959 ± 0.027	0.961 ± 0.023	0.960 ± 0.024	0.919 ± 0.049	0.864 ± 0.005	0.864 ± 0.009	0.865 ± 0.013	0.864 ± 0.006	0.729 ± 0.010
kNN	1.000 ± 0.000	1.000 ± 0.000	1.000 ± 0.000	1.000 ± 0.000	1.000 ± 0.000	0.764 ± 0.004	0.738 ± 0.003	0.820 ± 0.007	0.777 ± 0.004	0.532 ± 0.008
RF	1.000 ± 0.001	0.999 ± 0.001	1.000 ± 0.000	1.000 ± 0.001	0.999 ± 0.001	0.883 ± 0.013	0.866 ± 0.011	0.905 ± 0.018	0.885 ± 0.014	0.766 ± 0.027
Stack.	0.994 ± 0.004	0.994 ± 0.005	0.995 ± 0.003	0.994 ± 0.004	0.989 ± 0.008	0.893 ± 0.004	0.887 ± 0.005	0.900 ± 0.006	0.894 ± 0.004	0.786 ± 0.008

Descriptors

	Train Dataset					Test Dataset				
	Acc.	Prec.	Recall	F1	MCC	Acc.	Prec.	Recall	F1	MCC
SVC	0.926 ± 0.006	0.927 ± 0.005	0.925 ± 0.007	0.926 ± 0.006	0.852 ± 0.012	0.875 ± 0.004	0.860 ± 0.005	0.896 ± 0.004	0.878 ± 0.004	0.751 ± 0.008
LR	0.816 ± 0.001	0.807 ± 0.001	0.830 ± 0.002	0.818 ± 0.001	0.632 ± 0.002	0.843 ± 0.001	0.849 ± 0.002	0.835 ± 0.003	0.842 ± 0.001	0.687 ± 0.002
MLP	0.925 ± 0.008	0.923 ± 0.008	0.928 ± 0.009	0.926 ± 0.008	0.851 ± 0.016	0.860 ± 0.006	0.848 ± 0.007	0.878 ± 0.009	0.863 ± 0.006	0.721 ± 0.012
kNN	1.000 ± 0.000	1.000 ± 0.000	1.000 ± 0.000	1.000 ± 0.000	1.000 ± 0.000	0.845 ± 0.003	0.822 ± 0.004	0.882 ± 0.004	0.851 ± 0.003	0.692 ± 0.006
RF	0.999 ± 0.001	0.999 ± 0.002	0.999 ± 0.001	0.999 ± 0.001	0.998 ± 0.003	0.870 ± 0.004	0.863 ± 0.005	0.880 ± 0.006	0.871 ± 0.004	0.740 ± 0.008
Stack.	0.989 ± 0.003	0.992 ± 0.002	0.986 ± 0.003	0.989 ± 0.003	0.978 ± 0.005	0.880 ± 0.003	0.871 ± 0.003	0.891 ± 0.004	0.881 ± 0.003	0.759 ± 0.006
